# Evaluating malaria reactive surveillance and response strategies in northeast Cambodia: a mixed-methods study

**DOI:** 10.1186/s12936-025-05475-7

**Published:** 2025-07-13

**Authors:** Win Han Oo, Sovannaroth Siv, Kaungmyat Khant, Win Htike, Meach Monyth Molyta, Thet Lynn, Nilar Aye Tun, Paul A. Agius, Freya J. I. Fowkes

**Affiliations:** 1 Disease Elimination Program, Burnet Institute, Melbourne, VIC Australia; 2 Melbourne School of Population and Global Health, University of Melbourne, Melbourne, VIC Australia; 3 National Centre for Parasitology, Entomology and Malaria Control, Ministry of Health, Phnom Penh, Cambodia; 4 Health Security and Malaria Program, Burnet Institute Myanmar, Yangon, Myanmar; 5 Health Poverty Action, Phnom Penh, Cambodia; 6 Health Poverty Action, London, UK; 7 Faculty of Health, Deakin University, Melbourne, VIC Australia; 8 Department of Epidemiology and Preventive Medicine, Monash University, Melbourne, VIC Australia

**Keywords:** Cambodia, Malaria elimination, Mixed-methods study, Reactive surveillance and response strategy

## Abstract

**Background:**

Cambodia aims to eliminate malaria latest by 2030 applying the 1–3-7 malaria reactive surveillance and response (RASR) strategy which involves malaria case notification, investigation and classification on the same day as diagnosis, reactive case detection within three days, and investigation and classification of new active focus within seven days of case notification. This study investigates the implementation of the RASR strategy in terms of its timeliness, facilitators and barriers, and acceptability for implementation, thereby providing recommendations to improve the strategy in the context of the national health system.

**Methods:**

A mixed-methods study of secondary data analysis of aggregated routine malaria datasets, and cross-sectional survey, in-depth interviews and focus group discussions with malaria programme stakeholders, frontline health workers and mobile and migrant populations was conducted in Ratanakiri and Stung Treng provinces. Quantitative and qualitative data were analysed descriptively and thematically.

**Results:**

In 2020 and 2022, 72% and 59% of malaria cases were notified and investigated within one day after diagnosis. Timeliness of reactive case detection was 89% and 45% in 2020 and 2022 respectively. Despite having challenges including minimal community participation in reactive case detection, poor mobile phone network coverage and road conditions, a heavy workload at the commune health centre level, inadequate surveillance technical knowledge among village malaria workers and insufficient budget to execute RASR, the existing RASR strategy was deemed acceptable among all levels of health personnels.

**Conclusion:**

The RASR strategy implemented in northeast Cambodia was generally functioning well despite some challenges. To improve the RASR strategy to achieve 100% timeliness and progress towards malaria elimination in Cambodia, allocating sufficient budget, capacity building to frontline health workers and better community engagement strategies are required.

**Supplementary Information:**

The online version contains supplementary material available at 10.1186/s12936-025-05475-7.

## Background

Countries in the Greater Mekong Subregion (GMS) including Cambodia aim to eliminate malaria latest by 2030 and have made significant progress towards the goal over the past decades [1]. To achieve malaria elimination, national malaria programmes and their implementing partners are recommended by the World Health Organization (WHO) to convert malaria surveillance into a core intervention [2]. In a malaria elimination setting, case-based surveillance is critical to interrupt onward malaria transmission [3]. It comprises of (1) early detection of both asymptomatic and symptomatic cases, (2) prompt and adequate treatment of cases and vector control, and (3) focus investigation, classification and management to terminate ongoing transmission [4].

To execute effective case-based surveillance, the WHO recommends national malaria programmes to develop standard operating procedures with a schedule for reactive surveillance and response (RASR) strategies following the detection of a malaria case in elimination areas [4]. China became the first GMS country to receive malaria-free certification in 2021 by using the “1–3-7 RASR strategy” which involves case notification within one day, case investigation within three days, and focus investigation with appropriate public health responses within seven days after diagnosis of a malaria case [5]. Other GMS countries have adapted this strategy to improve their RASR activities to their own contexts advancing their goal towards malaria elimination [6].

Cambodia planned to eliminate all species of malaria in all the operational districts, an administrative unit for malaria elimination [7]. To effectively eliminate malaria, Cambodia adopted the 1–3-7 approach as its RASR strategy after pilot-testing in Sampov Loun operational district in 2015 for its implementation feasibility [8]. According to surveillance guidelines for malaria elimination by the National Centre for Parasitology, Entomology, and Malaria Control (CNM), the strategy involves 100% completeness and timeliness of case notification, investigation, and classification and reporting to the electronic Malaria Information System on the same day as diagnosis, reactive case detection (RACD) within three days of the notification and classification, and investigation and classification of every new active focus within one week [9].

To date, there has been no comprehensive and systematic evaluation of Cambodia’s RASR strategy in terms of its timeliness, acceptability, and facilitators and barriers to implementation. This mixed-methods study investigates how Cambodia implements its RASR strategy, its performance in terms of gaps in timeliness between the national standards and current implementation, facilitators and barriers, and acceptability and feasibility of implementation of the RASR strategy in its remote provinces, Ratanakiri and Stung Treng, to provide recommendations on how the strategy may be improved in the context of existing national health system.

## Methods

### Study design and settings

The mixed-methods study included secondary data analysis of aggregated datasets for timeliness of RASR activities reported between 1st January 2020 and 30th September 2022 (Additional file 1), quantitative cross-sectional survey, in-depth interviews (IDI) and focus group discussions (FGD) in Ratanakiri and Stung Treng Provinces in Cambodia.

**Fig. 1:**
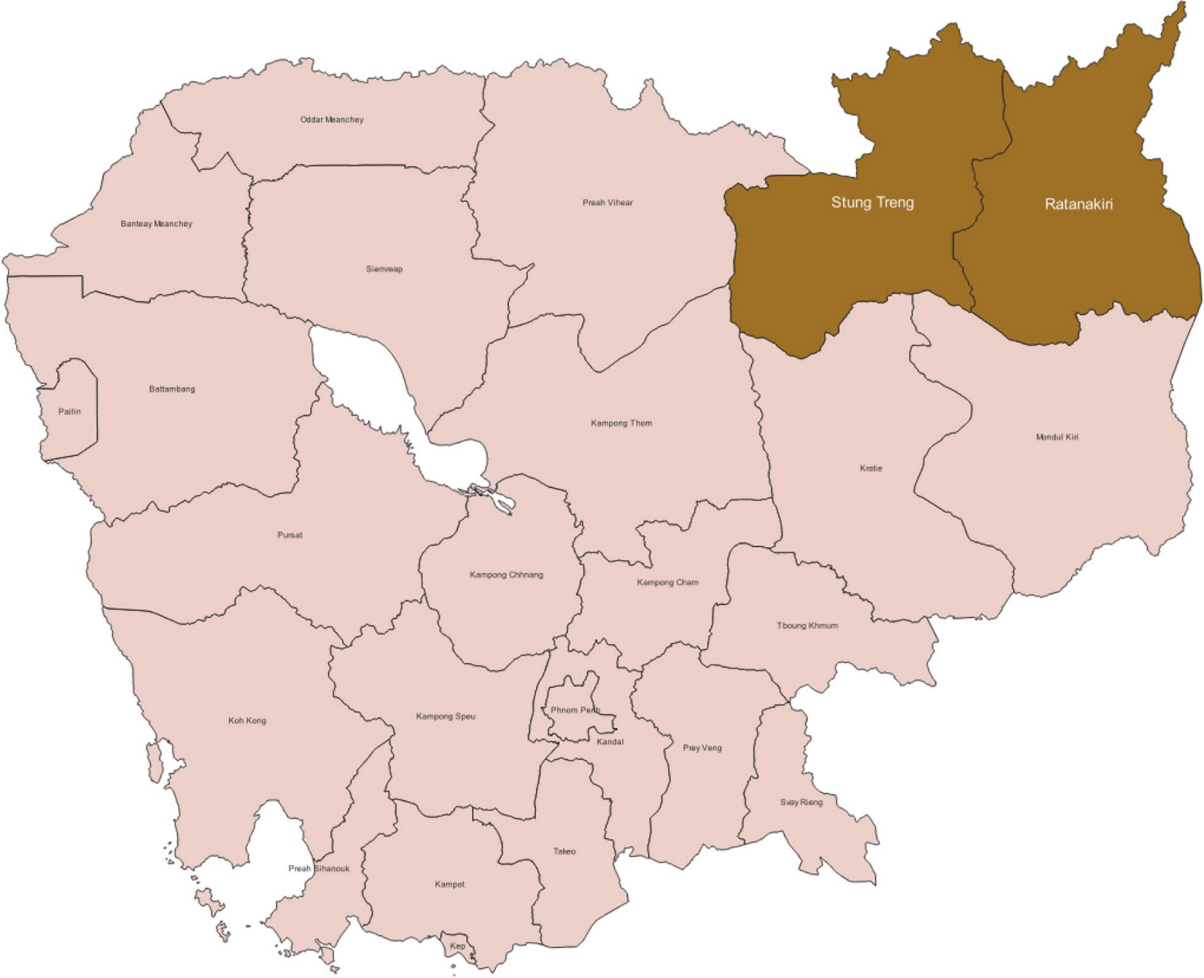
Map of the study areas. (Map generated using QGIS application version 3.22.0 using base maps from the Cambodia Subnational Administrative Boundary Common Operational Database (COD-SAB) developed by United Nations Office for the Coordination of Humanitarian Affairs downloaded from Humanitarian Data Exchange [14])

Ratanakiri and Stung Treng Provinces (Fig. 1**)** are remote provinces in northeastern Cambodia bordering Lao People’s Democratic Republic and Vietnam, characterised by tropical forests, rubber plantations and fallow lands. Ethnically diverse and home to mobile and migrant populations (MMPs), these provinces are among the highest risk areas for malaria in Cambodia [10]. According to the malaria intensification plan launched in November 2020, these provinces were committed to the elimination of falciparum malaria by 2023 [11] given annual parasite incidences in both provinces started to fall below 1 since 2021 [12] and became less than 0.1 in 2025 [13]. These provinces were selected in the study as per their malaria epidemiology, implementation status of the RASR activities and logistical feasibility.

### Data collection

The aggregated dataset for secondary data analysis was extracted from the National Malaria Surveillance Database of CNM on November 2, 2022. A cross-sectional survey was conducted using questionnaires (Additional file 2) with 40 malaria programme stakeholders and 40 frontline health workers (village malaria workers (VMW) and mobile malaria workers (MMW)), respectively (71 males, 9 females; Additional file 3, supplementary Table 1). Using FGD and IDI guides (Additional file 2), 11 FGDs with malaria supervisors, VMWs and forest goers (4 to 8 participants per FGD; 48 males, 11 females) and five IDIs with senior malaria stakeholders (4 males and 1 female of the national, provincial, and district levels) were conferred (Additional file 3, Supplementary Table 2).

Participants were selected purposively in consultation with CNM and in-country malaria implementing partners based on the criteria of having experiences with the RASR strategy and malaria program, and willingness to participate in the study.

All primary data collection happened in private locations where privacy was maintained. The survey, FGDs and IDIs were conducted in person in Khmer language between August 2022 and March 2023. Both IDIs and FGDs were audio recorded. Member checking with review and feedback from participants was applied during qualitative data collection to improve rigor. Data saturation was used as a cut-off point for ceasing qualitative data collection (FGD and IDI) while the survey enrolled all eligible participants in the areas who provided consent.

### Data management, analysis and reporting

From the secondary dataset, the percentages of timely malaria case notification and classification, RACD and focus investigation were calculated using the formulae: number of cases notified and classified on the same day as diagnosis divided by the total number of malaria cases, number of cases for which RACD was completed within three days divided by the total number of eligible cases for RACD, and number of cases for which focus investigation was completed within one week divided by the total number of eligible cases for focus investigation. The findings were multiplied by 100 to get the percentages.

The survey data were collected on paper forms, typed into REDCap platform, and formatted as per variables in the Questionnaire 1 and 2 (Additional file 2). The datasets were then imported into R version 4.2.1 [15] for analysis, including generating frequencies and percentages for categorical variables and calculating medians and interquartile ranges for numerical variables.

The audio records of IDIs and FGDs were transcribed verbatim in Khmer and then translated into English. They were organized, managed, immersed, and analysed thematically (deductive followed by inductive analysis) using NVivo software. Prior to coding, coding definitions and thematic frameworks were developed, discussed, and agreed. The coders bracketed out their personal experiences in data coding and analysis. After analysing data collected by each data collection method, the findings were triangulated to strengthen research evidence and reduce bias. The study is reported according to the Strengthening the Reporting of Observational Studies in Epidemiology (Additional file 4) and Consolidated criteria for Reporting Qualitative research (Additional file 5) checklists.

## Results

### Overall RASR strategy and policy

Almost all survey respondents (97.5%, 78/80) reported that their programme follows the 1–3-7 approach. Half (20/40) of the malaria programme stakeholders reported that this strategy was implemented across all areas of Cambodia, while the frontline health workers reported that it was applied only in malaria elimination areas (68%, 27/40; Additional file 3, Supplementary Table 3). FGD and IDI participants affirmed that they used 1–3-7 approach in case notification, investigation and classification, RACD and focus investigation of new active focus.

### Implementation and timeliness of RASR strategy

#### Case notification

A total of 2727 malaria cases were reported in Ratanakiri and Stung Treng Provinces between January 2020 and September 2022 out of which around 72% of cases in Ratanakiri and 59% of cases in Stung Treng were classified and notified on the same day as diagnosis in 2020 and 2022. Timeliness of case notification was lower in both Provinces in 2021 compared to 2020 and 2022 (42% in Ratanakiri and 36% in Stung Treng; Table 1). Survey participants also claimed that they completed the case surveillance report nearly always within the stipulated time frame (78%, 62/80) in 2020 and 2022 (Table 2).

**Table 1:** Timeliness of reactive surveillance and response activities in Ratanakiri and Stung Treng provinces between 2020 and 2022 (secondary data analysis)

	Notification and classification on the same day as diagnosis			Reactive case detection within three days			Focus investigation within one week		
	2020	2021	2022	2020	2021	2022	2020	2021	2022
	n (%)	n (%)	n (%)	n (%)	n (%)	n (%)	n (%)	n (%)	n (%)
Ratanakiri									
Cases	231	393	223	94	157	323	Not eligible	10	Not eligible
Completed timely	168 (72.7)	165 (42.0)	161 (72.2)	84 (89.4)	114 (72.6)	81 (89.0)	-	5 (50.0)	-
Stung treng									
Cases	725	497	658	253	181	232	21	11	16
Completed timely	428 (59.0)	177 (35.6)	390 (59.3)	116 (45.8)	43 (23.8)	103 (44.4)	19 (90.5)	2 (18.2)	15 (93.8)
Total									
Cases	956	890	881	347	338	323	21	21	16
Completed timely	596 (62.3)	342 (38.4)	551 (62.5)	200 (57.6)	157 (46.4)	184 (57.0)	19 (90.5)	7 (33.3)	15 (93.8)

**Table 2:** Timeliness of reactive surveillance and response activities (survey)

	Malaria programme stakeholders (n = 40)	Frontline health workers (n = 40)	Total (N = 80)
	n (%)	n (%)	n (%)
Frequency of case notification within one day of diagnosis			
Never	2 (5.0)	2 (5.0)	4 (5.0)
Occasionally (less than 20%)	3 (7.5)	1 (2.5)	4 (5.0)
More often than not (50–75%)	1 (2.5)	1 (2.5)	2 (2.5)
Usually (more than 75%)	5 (12.5)	3 (7.5)	8 (10.0)
Nearly always (more than 90%)	29 (72.5)	33 (82.5)	62 (77.5)
Time of initiation of case investigation after reporting a malaria case			
Within one day	33 (82.5)	27 (67.5)	60 (75.0)
Within two days	2 (5.0)	2 (5.0)	4 (5.0)
Within three days	2 (5.0)	10 (25.0)	12 (15.0)
Within one week	3 (7.5)	1 (2.5)	4 (5.0)
Time of initiation of foci investigation after reporting a malaria case			
Within one day	35 (87.5)	–	–
Within two days	0 (0)	–	–
Within three days	3 (7.5)	–	–
Within one week	2 (5)	–	–
Time of initiation of foci response after reporting a malaria case			
Within one day	28 (70.0)	30 (75.0)	58 (72.5)
Within two days	0 (0.0)	2 (5.0)	2 (2.5)
Within three days	8 (20.0)	8 (20.0)	16 (20.0)
Within one week	4 (10.0)	0 (0.0)	4 (5.0)
Completeness of case investigation			
For all cases (100%)	33 (82.5)	33 (82.5)	66 (82.5)
For > 75% of cases but < 100%	5 (12.5)	5 (12.5)	10 (12.5)
Between 50 and 75% of cases	1 (2.5)	1 (2.5)	2 (2.5)
Between 20 and 50% of cases	0 (0.0)	0 (0.0)	0 (0.0)
For < 20% of cases	1 (2.5)	1 (2.5)	2 (2.5)

According to surveys, almost all frontline health workers had access to mobile phone network (97.5%, 39/40) and internet (95.0%, 38/40). However, only about half of the frontline health workers (47%, 18/40) reported having access to reliable internet connectivity at their worksite or village for case notification (Additional file 3, Supplementary Table 4). Direct telephone calling to the respective staff was the most common case notification method among the frontline health workers (85%, 34/40), while many malaria programme stakeholders mentioned the electronic reporting system (80%, 32/40; Additional file 3, Supplementary Table 5).

#### Case investigation

Most surveyed malaria programme stakeholders (83%, 33/40) and frontline health workers (68%, 27/40) indicated that case investigation is initiated within one day of confirmation of an index malaria case (Table 2). Although many survey participants (83%, 66/80) (Table 2) suggested that all malaria cases had a case investigation completed, they also mentioned common reasons for incomplete case investigation such as cases moved to other districts and subsequently loss to follow-up (28%, 22/80), and inability to contact or reach the case (11%, 9/80; reasons detailed in Additional file 3, Supplementary Table 6).

*“There are villages located quite far (from the town/health centre. It is difficult to make a phone call or go there. This makes it challenging for the Team to conduct case investigations.” (A malaria stakeholder in FGD, Stung Treng province)*

The survey participants responded that case investigation was triggered by malaria cases reported to the national level (41%, 33/80) and peripheral level (68%, 54/80) of the malaria program. Many survey participants telephoned the index case to make an appointment for case investigation ahead of visiting to their home (65%, 52/80). If the case was not at home when they visited, they telephoned the index case again to schedule another appointment (76%,61/80) or visited the case again later the same day (58%, 46/80; Additional file 3, Supplementary Table 7).

Most frontline health workers in the survey were personally involved in case investigations (88%, 35/40). Many malaria programme stakeholders (73%, 29/40) reported that VMWs performed case investigations, while the remaining (28%, 11/40) reported that health centre staff or others performed it (64%, 7/11) (Additional file 3, Supplementary Table 8).

About two-thirds of the survey participants (66%, 53/80) claimed that they mapped the location of the index case during case investigation. Furthermore, a majority of participants in the survey (90%, 72/80) reported that they obtained travel history from the index case, including travel within (93.1%, 67/72) and outside of the district of residence (89%, 63/72), and outside of Cambodia (73%, 47/72) (Additional file 3, Supplementary Table 9). The malaria programme stakeholders in IDIs reported that they asked where the malaria-positive person had been sleeping two weeks before, and who were the co-travellers.

Based on the travel history, they classified the malaria case as either (1) local/within-village transmission, (2) out-of-village transmission, (3) imported case, (4) or relapse/recurrence. Of the survey participants, 68% (54/80) defined imported cases as cases originating from another country, while the remaining respondents (33%, 26/80) defined imported cases as cases from another district within the country (Additional file 3, Supplementary Table 9). A staff in IDI explained the four classes of malaria cases.

*“There are four types of malaria cases. The first type, L1, is cases where the origin of the case is local from the patient's village of residence as defined by the fact that the patient has slept every night at their village of residence within the last 2 weeks. LC is also local cases, but this time the patient has slept at least one night outside of their village of residence but within Cambodia over the past two weeks. For imported cases called IMP, the transmission origin is from another country and the criteria is that the patient has slept at least one night in another country within the last two weeks. Lastly, there's a type called REL/REC for cases from a previous episode of malaria, where the patient has recent P. vivax infection and has had P. vivax within the last 12 months”. (A malaria stakeholder in IDI, Stung Treng province)*

Most survey participants (89%, 71/80) reported that there was periodic supervision on conducting case investigations, which could be monthly (54%, 38/71), quarterly (20%, 14/71), or yearly (10%, 7/71). The VMWs and MMWs reported that they were supervised during case investigations by staff from a health centre (79%, 30/38), district health department (29%, 11/38), provincial health department (13%, 5/38) and Catholic Relief Services (11%, 4/38; Additional file 3, Supplementary Table 10).

All 80 survey respondents reported that the individuals conducting case investigations had received case investigation training organised monthly (18%, 14/80), quarterly (8%, 6/80), yearly (53%, 42/80), or biennially (23%, 18/80; Additional file 3, Supplementary Table 9). Concurrently, VMWs from the FGDs stated that trainings related to 1-3-7 approach, including case investigation and RASR guidelines were provided to them by the national malaria programme, provincial health department, their partner organizations and health centres.

### Reactive case detection

Between 2020 and 2022, 1,008 malaria cases (60 imported or out-of-village transmitted cases of *Plasmodium falciparum* or mixed species and 948 within-village transmitted *Plasmodium vivax* cases) were eligible for RACD; about half of the cases (54%, 541/1008) had RACD carried out within three days of index case classification. The timeliness of RACD was similar in 2020 and 2022 but varied according to province (Ratanakiri, 89%; Stung Treng, ~ 45%) and was lowest in 2021 (73% and 24% in Ratanakiri and Stung Treng respectively) (Table 1).

Almost all surveyed malaria programme stakeholders (92.5%, 37/40) reported that their malaria elimination programme practiced RACD using rapid diagnostic test (RDT) (100%, 80/80). Other tools used in RACD included microscopy (15%, 12/80), polymerase chain reaction (PCR) (2.5%, 2/80) and serology (1.2%, 1/80) (Table 3).

**Table 3:** Reactive case detection (survey)

	Malaria programme stakeholders (n = 40)	Frontline health workers (n = 40)	Total (N = 80)
	n (%)	n (%)	n (%)
Does your programme routinely conduct reactive case detection (RACD)?			
No	3(7.5)	–	
Yes	37(92.5)	–	
When conducting RACD, which diagnostic tool is used?			
Rapid diagnostic test	40 (100.0)	40 (100.0)	80 (100.0)
Microscopy	8 (20.0)	4 (10.0)	12 (15.0)
Polymerase chain reaction	1 (2.5)	1 (2.5)	2 (2.5)
Serology	1 (2.5)	0 (0.0)	1 (1.2)
What triggers screening in the community?			
Local case only	21 (52.5)	18 (45.0)	39 (48.8)
Local and imported cases	19 (47.5)	11 (27.5)	30 (37.5)
Imported cases only	0 (0.0)	1 (2.5)	1 (1.2)
When local cases reach a minimum threshold	0 (0.0)	10 (25.0)	10 (12.5)
Median threshold number of local cases (IQR)	–	5 (5 to 9)	5 (5 to 9)
Do you screen household members of the index case?			
Always	35 (87.5)	39 (97.5)	74 (92.5)
Never	1 (2.5)	1 (2.5)	2 (2.5)
Sometimes	4 (10.0)	0 (0.0)	4 (5.0)
When screening, which household members do you screen?			
Febrile cases only	2 (5.1)	4 (10.2)	6 (7.7)
All household members (asymptomatic and febrile cases)	37 (94.9)	35 (89.7)	72 (92.3)
When screening neighbours of the index case, are febrile individuals tested or all individuals?			
Febrile neighbours only	9 (22.5)	6 (15.0)	15 (18.8)
All neighbours	31 (77.5)	34 (85.0)	65 (81.2)
Do you screen a minimum number of households around a positive index case?			
No	1 (2.5)	1 (2.5)	2 (2.5)
Yes	39 (97.5)	39 (97.5)	78 (97.5)
If “Yes”, please specify minimum number of households			
Median (IQR)	20 (20 to 20)	20 (20 to 23.8)	20 (20 to 20)
Range	20 to 25	3 to 40	3 to 40
Do you screen within a minimum geographic radius around a positive index case?			
No	6 (15.0)	8 (20.0)	14 (17.5)
Yes	34 (85.0)	32 (80.0)	66 (82.5)
If “Yes”, please specify the radius			
Within one kilometre	28 (82.4)	27 (84.4)	55 (83.3)
Within 2–5 kms	6 (17.6)	4 (12.5)	10 (15.2)
More than 5 kms	0 (0.0)	1 (3.1)	1 (1.5)

Nearly half of the survey participants (49%, 39/80) reported that RACD was triggered only by local cases. Less than half of malaria programme stakeholders (48%, 19/40) and only about a quarter of VMWs and MMWs (28%, 11/40) responded that screening for RACD was triggered by local and imported cases (Table 3). In IDIs, a malaria programme stakeholder reported that RACD was triggered by imported and domestic *P. falciparum* or mixed cases and within-village transmitted *P*. *vivax* cases.

*“Reactive case detection was performed for cases of Plasmodium falciparum or mixed infection found as domestic cases (LC) or imported malaria (IMP), and village cases for Plasmodium vivax cases (L1).” (A malaria stakeholder in IDI, Stung Treng)*

Majority of the survey participants reported that RACD always screened the household members of the index cases (92.5%, 74/80) and included all the household members (92.3%, 72/78). They reported that RACD also included screening both asymptomatic and febrile neighbours (81%, 65/80; Table 3). The median number of households screened was 20 (min–max: 3–40, n = 78). Most survey participants reported that minimum screening radius was within one kilometre around a positive index case (83%, 55/80; Table 3). IDIs revealed that co-travellers of the index cases were also tested during the RACD.

*“The activities of reactive case detection include testing for malaria among all members of the index case household and 20 neighbouring households, or people who reside within one kilometre around the index case and also testing for malaria among co-travellers who may miss in the initial testing by geographical criterial.” (A malaria stakeholder in IDI, Stung Treng province)*

### Focus investigation and responses

Secondary data analysis revealed that the majority of the eligible foci with local *P. falciparum* or mixed cases were investigated and classified within one week (71%, 41/58) after an index malaria case had been reported. Notably, there were zero eligible cases for focus investigation in Ratanakiri Province in 2020 and 2022. In 2021, exactly half of the foci in Ratanakiri (50%, 5/10) were investigated within seven days. Stung Treng province achieved > 90% timeliness in focus investigation in 2020 and 2022 but was lower in 2021 (18%, 2/11) (Table 1).

Malaria programme stakeholders in the survey reported that focus investigation was initiated within one day (88%, 35/40), three days (7.5%, 3/40) or one week (5%, 2/40), respectively, after a malaria case was reported (Table 2). A programme staff explained in an IDI that focus investigation was triggered by confirmed cases of within-village transmitted *P. falciparum* or mixed infection, and it was expected to be completed within seven days.

Based on the survey findings, focus response began within one day after a malaria-positive case had been reported (73%, 58/80) (Table 2). The most common response activity among the survey participants was raising awareness about malaria transmission (93.8%, 75/80), while frontline health workers also reported malaria prevention (75%, 30/40) and additional vector control activities (60%, 24/40) as focus responses (Additional file 3, Supplementary Table 11).

Notably, both malaria programme stakeholders and frontline health workers in the survey thought that information gained from case and focus investigations did not influence the response activities (71%, 57/80). Furthermore, the malaria programme stakeholders believed that the current RASR activities were not sufficient to target MMPs (53%, 21/40) and *P. vivax* malaria (63%, 25/40) (Additional file 3, Supplementary Table 12).

### Acceptability and feasibility of RASR strategies

#### Acceptability of RASR strategy

According to FGDs and IDIs, programme stakeholders expressed satisfaction with the existing RASR strategy and cited positive outcomes in the malaria elimination programme associated with the strategy. In addition, the VMWs were also satisfied with their increased opportunities to participate in surveillance activities and works with the malaria programme stakeholders. Moreover, MMPs participating in the FGDs expressed their desire to see the RASR activities continue in their communities. Overall, the current RASR strategy was acceptable across all levels of malaria stakeholders, frontline malaria providers and community members.

*“The current malaria RASR strategy and activities are acceptable to different malaria programme stakeholders at different levels across various geographical areas because we see a better result (in the malaria elimination program) after initiation of the 1-3-7 strategy.” (A malaria stakeholder in IDI, Ratanakiri province)*

#### Feasibility of RASR strategy

The FGDs and IDIs data highlighted several factors that support the feasible implementation of the RASR strategy in Cambodia. A senior officer in IDI mentioned the “Malaria Eradication Commission”*,* which has extended structure from community to national level. The committee secured improved support and commitment from the politicians and the government to the RASR strategy. In addition, both the malaria programme stakeholders and VMWs committed to the RASR strategy no matter what the circumstances given their dedication and hard work contributed to the success of the RASR strategy. Moreover, a district level malaria stakeholder in an IDI believed that the involvement of local authorities with great influence on the community participation in the surveillance activities, was a key to successful implementation of RASR strategy. They also cited the resources support of partner organisations as a helpful factor in successful implementation of RASR activities.

*“The reason why RASR strategy is working is because of the hard work and dedication of malaria programme stakeholders and volunteers (VMWs), who are committed to the (RASR) strategy, no matter what the circumstances.” (A malaria stakeholder in IDI, Ratanakiri province)*

Furthermore, malaria supervisors in FGDs also claimed that there were adequate commodities and human resources to implement the RASR strategy. They stated that the Ministry of Health and its partner organisations supplied the materials monthly and the district health department would supply the required commodities once they requested them in a report. They also felt that the human resources available through the district and community health centre, partner organisations, VMWs and MMWs were sufficient to implement the RASR strategy. A malaria programme stakeholder in the IDI believed that the guidelines for case classification and schedule in the current RASR strategy were well-suited to existing national health system and infrastructure as well as the socio-cultural backgrounds.

### Challenges for successful implementation of RASR strategy

Although the RASR strategy is acceptable and feasible to implement generally, and many of the survey participants (63%, 50/80) reported that there was no barrier to follow the guidelines of RASR activities, there were some barriers to its implementation. Poor mobile phone connection and internet access were the main barriers to timely case notification (65%, 52/80), case investigation (39%, 31/80) and focus investigation (20%, 16/80). In addition, poor community participation (51%, 41/80) and transportation difficulties (28%, 22/80) were the most cited challenges in carrying out RACD (Table 4). The COVID-19 pandemic also had impact on implementation of the RASR strategy (45%, 36/80) (Additional file 3, Supplementary Table 12). The survey results were supported by qualitative findings which identified two primary categories of obstacles to RASR strategy: health system challenges such as limitations in human and financial resources, and operational challenges such as COVID-19 pandemic, poor community participation, migration of people, telecommunication problems, and transportation difficulties.

**Table 4:** Barriers to implementing reactive surveillance and response strategies (survey)

	Malaria programme stakeholder (n = 40)		Frontline health workers (n = 40)		Total (N = 80)
	n (%)		n (%)		n (%)
What are the barriers to timely case notification?					
Difficult communication (phone or internet)		30 (75.0)	22 (55.0)		52 (65.0)
Transportation difficulty		5 (12.5)	5 (12.5)		10 (12.5)
Human resource problem		2 (5.0)	1 (2.5)		3 (3.8)
Poor community involvement		0 (0)	2 (5.0)		2 (2.5)
No barrier		9 (22.5)	16 (40.0)		25 (31.2)
What are the barriers to timely case investigation?					
Difficult communication (phone or internet)		15 (37.5)	16 (40)		31(38.8)
Transportation difficulty		6 (15.0)	3 (7.5)		9 (11.2)
Late case notification		4 (10.0)	0 (0)		4 (5.0)
Loss to follow-up		3 (7.5)	0 (0)		3 (3.8)
No barrier		17 (42.5)	24 (60.0)		41 (51.2)
What are the challenges for RACD?					
Poor community involvement		17 (42.5)	24 (60.0)		41 (51.2)
Transportation difficulty		11 (27.5)	11 (27.5)		22 (27.5)
Can not meet with stakeholders		9 (22.5)	0 (0)		9 (11.2)
Difficult communication (phone or internet)		1 (2.5)	6 (15.0)		7 (8.8)
Budget constraint		2 (5.0)	3 (7.5)		5 (6.2)
Human resource problem		0 (0)	1 (2.5)		1 (1.2)
No challenge		10 (25.0)	3 (7.5)		13 (16.2)
What are the barriers to timely foci investigation and response?					
Difficult communication (phone or internet)		10 (25.0)		6 (15.0)	16 (20)
Transportation difficulty		3 (7.5)		5 (12.5)	8 (10.0)
Index case does not get medication according to guidelines		2 (5.0)		2 (5.0)	4 (5.0)
Late case notification		3 (7.5)		0 (0)	3 (3.8)
Lack of responsibility among some VMWs and MMWs		2 (5.0)		0 (0)	2 (2.5)
Human resource problem		0 (0)		1 (2.5)	1 (1.2)
Migrant population		1 (2.5)		0 (0)	1 (1.2)
No barrier against		23 (57.5)		29 (72.5)	52 (65.0)
What are the barriers to following guidelines for implementing RASR activities?					
Difficult communication (phone or internet)		13 (32.5)	7 (17.5)		20 (25)
Transportation difficulty		7 (17.5)	1 (2.5)		8 (10)
Poor community involvement		4 (10.0)	2 (5.0)		6 (7.5)
Don’t report and follow-up on time		0 (0)	2 (5.0)		2 (2.5)
Poor knowledge of guideline		0 (0)	2 (5.0)		2 (2.5)
Not much time for implementation of activities		0 (0)	1 (2.5)		1 (1.2)
No responsibility		0 (0)	1 (2.5)		1 (1.2)
No barrier		21 (52.5)	29 (72.5)		50 (62.5)

### Health system challenges

In contrast with malaria supervisors from FGDs who cited having enough human resource as an enabler, a malaria stakeholder in IDI pointed out that human resource shortages at the health centres were a challenge for successful implementation of RASR activities. The health centre staff were responsible not only for malaria but also for other disease programs, and thus, one staff had to work on multiple tasks simultaneously*.* Heavy workload at the commune level led to delays in completing the RASR activities. Additionally, the IDIs revealed that some VMWs did not have adequate technical knowledge. They could not carry out malaria testing and complete the required documentations because of their limited knowledge on the subject matter and information technology.

Malaria supervisors and VMWs reported in FGD that financial support they received was not enough to conduct RASR activities especially for screening all the suspected households around the index case’s residence during RACD. Furthermore, the VMWs and MMWs were only rewarded with minimal incentives, receiving only three United States dollars per malaria case reported, but no incentive for negative RDT tests conducted. Additionally, increasing costs of gasoline and motorcycle maintenance required for travelling to the remote villages were not covered by the Programme. Besides, VMWs and MMWs had to work to earn a living, occasionally resulting in less effort with RASR activities.

*“When a malaria case is detected in a community, it requires investigating and screening the population surrounding the index case. The challenge is insufficient budget to do so.” (A malaria stakeholder in FGD, Ratanakiri province)*

### Operational challenges

FGDs revealed that community members were confusing between COVID-19 and malaria screenings, and this made RASR activities difficult to implement. Some community members were afraid of COVID-19 infection and refused to meet the malaria programme stakeholders conducting RASR activities. Due to COVID-19 control measures, RASR activities could only be implemented among the malaria high-risk groups because of resources limitation and social distancing.

Poor community participation was identified as a significant obstacle to RASR activities during FGDs and IDIs. Reaching all individuals eligible for RACD was challenging because they worked in remote areas such as forests or farms. VMWs and MMWs could not follow them to their worksites and had to wait for their return, causing significant delays in completing RASR activities. Additionally, the lack of cooperation from some local authorities significantly impacted the malaria programme stakeholders' ability to announce, communicate and conduct RASR activities in the communities.

*“During reactive case detection, the people to be screened (for malaria) are often not at home as they are working in their farms or forests from morning until evening.” (A malaria stakeholder in IDI, Stung Treng province)*

According to IDIs, unpredictable mobility patterns of MMPs made mapping their location in conducting RACD and contacting them for treatment difficult, hindering completion of RASR activities in time. Furthermore, the lack of information on the size of MMP population challenged malaria testing and treatment and distributing mosquito net. Additionally, many MMPs had poor malaria knowledge and disregarded RASR activities resulted in refusing to cooperate with screening efforts. This, coupled with the lack of support from employers of MMPs, made screening at the workplace even more difficult.

*“Challenges in implementing RASR activities among MMPs include difficulty in carrying out active case detection due to their rapid displacement. Monitoring treatment and drug distribution before they enter into forest are also challenging.” (A malaria stakeholder in IDI, Stung Treng province)*

FGDs also highlighted difficult transportation that hindered the timely execution of RASR activities. The poor road conditions in malaria-endemic remote areas made it difficult to travel by motorbike to conduct RASR activities. It was more common during the rainy season when flooding was common, and motorbikes broke down frequently. It made the health personnel’s travelling time longer resulting in delayed RASR activities.

*“In remote areas, the condition of the roads can be a significant challenge for us, particularly during the rainy season. During this time, even a short distance of 5-10 km can become a long journey taking 1 to 2 hours to drive.” (A malaria programme stakeholder in FGD, Ratanakiri province)*

Moreover, limited mobile phone and internet signals at malaria endemic areas was a major barrier to timeliness of RASR activities, especially case notification. The loss of internet connection caused errors while reporting malaria cases to the Malaria Information System, rendering case notification incomplete. As a result of the limited mobile phone network, malaria cases had to be reported on the second day of diagnosis, resulting in delayed case notification.

*“In areas where mobile phone network is unavailable, such as in some villages and farms, it can be challenging for VMWs to contact cases or notify positive cases to the programme staff.” (A malaria stakeholder in IDI, Ratanakiri province)*

## Discussion

This study evaluated the adherence of each step of RASR activities to the 1–3-7 schedule, assessed the perception and practice of the RASR strategy by malaria programme stakeholders and frontline health workers, and explored the enablers and barriers to the implementation of the RASR strategy in Ratanakiri and Stung Treng provinces of Cambodia. There were delays in case classification and notification in about 30–40% of cases as well as RACD and focus investigation in 2020 and 2022 which was amplified in 2021. Study participants acknowledged Cambodia’s adoption of the 1–3-7 approach and found it to be acceptable and feasible to implement in the study provinces. Nevertheless, poor community participation, insufficient financial support, telecommunication problems for reporting and contacting cases, and transportation difficulties in remote areas were identified as major challenges to successful implementation of the RASR strategy. These barriers must be addressed so that the timely execution of each step of RASR will improve and achieve 100%, a global standard in the malaria elimination programme, which will ultimately contribute towards achieving national and regional malaria elimination goals.

### Case notification

Case notification is the first and important step for completing the whole continuum of RASR activities in a timely manner. Profound outbreaks of COVID-19 in Cambodia in 2021 [16] disrupted malaria surveillance and response activities, resulting in a significant drop in timely notification rates in 2021 compared to those of 2020 to 2022. Lockdowns due to COVID-19 outbreaks, coupled with a lack of reliable mobile network in remote areas, caused delays in VMWs and MMWs notification of malaria cases to the health centres and the Malaria Information System [17]. Ratanakiri reported higher timely notification rates than Stung Treng throughout the study period, which may be due to differences in provincial health system and infrastructures. Siem Pang district in Stung Treng province is malaria-endemic and had to implement many RASR activities but there were only two health centres for the whole district [18] responsible for RASR activities. Therefore, the resources and infrastructure were not sufficient to provide malaria services to the whole district, leading to lower timeliness in the RASR activities including case notification. This highlights the need for strong healthcare infrastructure across all provinces to facilitate the implementation of RASR in Cambodia.

According to the survey results, there was a difference in reporting methods used to notify malaria cases between malaria programme stakeholders and frontline health workers. Unlike malaria programme stakeholders who mentioned using the electronic reporting system, frontline health workers preferred telephone calling for case notification. Similarly in Vietnam, another GMS country, frontline health workers initially reported malaria cases mainly via telephone calling [19]. One possible reason is the limited internet access but good mobile phone network coverage in the endemic remote areas causing MMWs demotivated to use the electronic reporting system, making the telephone calling method the most feasible option.

Electronic reporting system can streamline the RASR activities and helps achieving malaria elimination [20]. Thus, the utilization of the electronic reporting system for initial case notification should be promoted among the VMWs and MMWs which demands a good internet connection in remote malaria endemic areas. Therefore, multisectoral approach is necessary to increase the coverage of good internet connection in the whole country. Another solution is to incentivise the VMWs and MMWS to go to the nearest area that has good internet coverage to report each malaria case within one day after diagnosis.

### Case investigation

In 2021 CNM issued a guideline to undertake case investigation and classification at the point of care within one day by the provider who diagnosed malaria typically VMW or MMW, which is different from other countries in GMS [9] where health centre staff perform case investigaiton within 3 day. However, there can be limitations to this approach because of the capacity of the persons conducting case investigation. Case investigation is a complex procedure and even challenging for the health centre staff to correctly execute in time [21]. Moreover, case classification could be subjective depending on the experience of the health personnel and the information received from the index case [22]. Accurate classification of a malaria case also requires a sound knowledge of malaria epidemiology [22–24]. Although CNM has established a clear and consistent definition of imported case to ensure uniformity in case classification, some participants in the survey did not know the definition. Moreover, qualitative findings highlighted low technical competency regarding case classification among the VMWs and MMWs. This finding highlighted the need for information sharing, frequent training and capacity building of VMWs to ensure consistent adherence to guidelines and more reliable case classification.

A major reason for incomplete case investigation was loss to follow-up on cases moving to districts outside of the VMW’s assigned district. Frequent and unpredictable migration, and telecommunication and transportation difficulties might lead to loss to follow-up in GMS [22, 25, 26]. Hence, improved communication and coordination between districts and implementation of an appointment system to effectively investigate and treat malaria cases may benefit case investigation in terms of completeness and timeliness.

In the fight against malaria, empowering communities and community volunteers is critical [2]. Frontline health workers such as VMWs and MMWs showed their commitment to case investigation. Their ability to complete preliminary case investigation within the given time schedule can be an advantage for accessibility to malaria services and promptly carrying out responses to interrupt malaria transmission [27, 28]. Hence, it is essential to improve the capacity of VMWs and MMWs to ensure the effectiveness and efficiency of case investigation.

Regular training in case investigation techniques reinforces technical skills of VMWs and MMWs to adapt the changing epidemiology of malaria in the elimination setting [29, 30]. Receiving RASR training biennially was the second highest in the survey, reflecting the challenges associated with providing regular training in remote or hard-to-reach areas where the workload may be heavy and access to resources and infrastructure may be limited. In this resource limited setting, ‘meeting plus training’ format will save the programme’s resources and time and improve the training frequency [30].

Geographic mapping of the index case location during the case investigation helps to identify the area of malaria transmission, predict the risk of transmission in hotspots along the MMP movements [31, 32], facilitating the RACD, focus investigation and responses [3]. However, the unpredictable mobility patterns of the MMPs are still hindering mapping the location of the index case. To handle this, CNM has assigned MMWs to visit the MMP clusters near their villages twice a month and test at least 20 MMPs per visit [9]. This will improve the efficient use of resources in targeted malaria elimination programme. Therefore, MMWs should be able to collect geolocation of malaria cases using their smartphone during case investigation.

### Reactive case detection

There was suboptimal performance of RACD during the study period, with only around 54% of RACD being undertaken in a timely manner. Poor community participation in screening for malaria cases, and transportation difficulties to reach remote high-risk populations hindered RACD implementation, necessitating targeted interventions to improve RACD timeliness [19, 21, 22].

Community participation in RACD is a vital factor in the success of the RASR strategy for malaria elimination [33]. To improve community involvement in RACD, better approaches for community engagement need to be considered. One possible solution is empowering and engaging community members in planning and executing the RASR strategy using participatory methods [27, 34, 35]. From this study’s perspective, community authorities played a critical role in community engagement as they could influence the community members [34, 35], and hence community authorties could be used to improve community engagment for RACD.

CNM recommends using RDT for RACD, and survey findings suggest that all participants mentioned RDTs as the primary diagnostic tool in conducting RACD, showing compliance with the guidelines [9]. However, RDT has limited sensitivity in detecting subclinical malaria infections [36] and, therefore, more sensitive molecular diagnostic methods such as PCR should be included in the surveillance guidelines of CNM [9]. In executing RACD, PCR showed higher case yield than RDT and microscopy in a meta-analysis study in the GMS [37]. Relying solely on RDT can miss asymptomatic malaria infections, hindering malaria elimination efforts. However, in resource-limited countries, RDT and microscopy are the only affordable options for RACD because molecular diagnostic tools including PCR are expensive, require skilled workers and specialized infrastructure, and are not sustainable for long-term use [33]. In this context, the country specific RACD tool could be set balancing the tool’s sensitivity and feasibility.

Cambodia’s guidelines for RACD recommend triggering RACD in response to both imported and locally acquired cases [9]. However, the survey results showed that both the malaria programme stakeholders and VMWs had limited knowledge about the triggers for RACD. This could have significant implications for the effectiveness of RACD in elimination setting. Triggering RACD only in response to local cases may have missed imported *P. falciparum* cases and hindered the effort to eliminate *P. falciparum* in 2023 [38, 39]. On the other hand, activating RACD for both local and imported *P. vivax* cases could be resource intensive.

The guidelines for RACD in Cambodia recommend testing neighbours of index case if they have fever with chills and rigors, and having previous malaria history, malaria case(s) in the household or risk factors such as having forest trip or travelling to high-risk malaria areas in the past 30 days. However, a majority of the survey participants screened all neighbours during RACD, regardless of the presence of risk factors. This could result in ineffective targeting of high-risk areas and individuals and reduce the overall effectiveness including case yield of RACD [40]. Additionally, this practice could lead to an increase consumption of resources and decrease community trust.

### Focus investigation and responses

There were differences in response patterns reported by malaria programme stakeholders and frontline health workers, with the former focusing mainly on raising awareness about malaria transmission while the latter emphasized prevention, vector control, and treatments as important response activities. One possible reason is that programme stakeholders focused on the bigger picture of malaria transmission while the frontline health workers emphasized the practical aspects of focus response. This difference indicates that there are variations in priorities between these two groups in addressing malaria focus, which could have implications on the effectiveness of focus response activities. This calls for more comprehensive and collaborative approaches between these two groups in focus response activities.

### Acceptability and feasibility of RASR strategy

The existing RASR strategy in Cambodia was perceived as feasible to implement by various stakeholders in the malaria programme due to the involvement of dedicated VMWs and MMWs, and local authorities, effective RASR training, and sufficient resources provided by the government and partner organisations: common factors for feasibility of scaling up public health interventions [41]. Its compatibility with the national context could result in successful malaria elimination in Cambodia.

### Strength and limitations

This evaluation is the first study in Cambodia examining the adherence of the RASR strategy to the 1–3-7 schedule and the perception among malaria programme stakeholders, VMWs and MMWs on the RASR strategy. The study utilised various data collection tools and methods tailored to each group of participants, and findings were triangulated to reinforce validity. However, the study was conducted only in Ratanakiri and Stung Treng provinces and generalisation of the study findings in other provinces should be made with caution. Furthermore, only the aggregated secondary dataset that lacks individual line lists of malaria patients was extracted from Malaria Information System and analysed. Thus, factors influencing the timeliness of the RASR activities could not be further explored. Another important indicator of RASR strategy, the completeness, couldn’t be calculated from the secondary dataset that compromises the information on overall coverage of RASR activities in Cambodia.

## Conclusion

Overall, the RASR activities in Cambodia were executed timely despite some delays in each step caused by transportation and communication issues. It is acceptable to stakeholders and frontline health workers in northeast Cambodia and may be feasible to implement in the national health system context. To further improve the performance and effectiveness of the RASR strategy in Cambodia and contribute towards the goal of regional malaria elimination, a set of recommendations (Table 5) is proposed.

**Table 5:** Summary of recommendations to improve reactive surveillance and response strategies

Activities	Issues	Recommendations
Case notification	• Delaying due to lack of or weak mobile phone signal and internet access • The preference of VMWs and MMWs with telephone calling over the electronic reporting system	• Incentivising VMWs and MMWs to travel to the nearest internet accessible area and notify the cases in time • Capacity building and incentivising VMWs and MMWs to use the electronic reporting system
Case investigation	• Limitation in case investigation and classification knowledge and skills of VMWs and MMWs • Incomplete case investigation and management due to loss to follow-up of patients • Gap in mapping the location of MMP index cases	• Organising trainings in ‘meeting plus training’ format for VMWs and MMWs • Developing a better appointment system for follow-up across the districts • Capacity building to MMWs to instantly collect GPS data of the index case during their first visit to MMPs
RACD	• Poor community participation in malaria screening • Inadequate travel allowance for executing RACD • Limitation of RDT in detecting subclinical malaria • Deviation from RACD guidelines	• Targeted counselling to community stakeholders for better community engagement • Revising the travel allowance policy as per the updated market prices • Piloting new diagnostic tools such as next generation RDTs • Providing frequent refresher trainings and communicating guidelines to VMWs and MMWs
Focus investigation	• Different focus response patterns mentioned by stakeholders and VMWs/MMWs	• Establishing comprehensive and collaborative approaches of focus response activities
Overall	• Unattractive incentives for frontline health workers • Terrain difficulties • Challenges of reaching out and treating MMPs	• Revising RASR incentive scheme and closed supervision to motivate frontline health workers • Capacity building, empowerment and task shifting to VMWs and MMWs in hard-to-reach areas • Engaging employers of MMPs

*GPS* Global Positioning System, *MMP* Mobile Migrant Population, *MMW* Mobile Malaria Worker, *VMW* Village Malaria Worker, *RACD* Reactive Case Detection, *RASR* Reactive Surveillance and Responses, *RDT* Rapid Diagnostic Test

## Supplementary Information

Additional file 1. Secondary aggregated dataset for timeliness of reactive surveillance and response activities in Ratanakiri and Stung Treng Provinces

Additional file 2. Questionnaire 1 and 2 to survey malaria programme stakeholders and frontline malaria service providers, and focus group discussion and in-depth interview topic guides for qualitative data collection

Additional file 3. Supplementary tables

Additional file 4. Strengthening the Reporting of Observational Studies in Epidemiology checklist

Additional file 5. Consolidated criteria for Reporting Qualitative research checklist

## Data Availability

The secondary aggregated dataset analysed in this study is included as Additional file 1. Survey and qualitative datasets analysed in this study are available from the corresponding author on reasonable request.

## Abbreviations

CNM: National Centre for Parasitology, Entomology and Malaria Control
FGD: Focus group discussion
GMS: Greater Mekong Subregion
IDI: In-depth interview
MMP: Mobile and migrant population
MMW: Mobile malaria worker
PCR: Polymerase chain reaction
RACD: Reactive case detection
RASR: Reactive surveillance and response strategy
VMW: Village malaria worker
WHO: World Health Organization
