# Supplementary material for: Evaluating malaria reactive surveillance and response strategies in northeast Cambodia: a mixed-methods study
Source: Malar J. 2025 Jul 13;24:229. doi: 10.1186/s12936-025-05475-7 (PMC12255966; doi:10.1186/s12936-025-05475-7)
Supplement: Supplementary file 2 — Additional file 2. Questionnaire 1 and 2 to survey malaria programme stakeholders and frontline malaria service providers, and focus group discussion and in-depth interview topic guides for qualitative data collection [file 12936_2025_5475_MOESM2_ESM.docx]

### Additional file 2 – Data collection tools

### Questionnaire 1: Malaria program stakeholders responsible for managing or supervising field reactive surveillance and response activities

| **General background Information** | | | |
| --- | --- | --- | --- |
| 0.1 | Date (DD/MM/YYYY): | |  |
| 0.2 | Country: | |  |
| 0.3 | Name of organization | |  |
| **Background information of Malaria Program Stakeholder (manager/supervisor of field activities)** | | | |
| 0.4 | Completed age (in years): |  | |
| 0.5 | Gender | 1. Male 2. Female 3. Other/prefer not to say | |
| 0.6 | Highest education level | 1. No formal education 2. Primary school level 3. Secondary school level 4. High school level 5. Degree holder 6. Others (specify) | |
| 0.7 | What is your current role |  | |
| 0.8 | How long have you worked in your current role | ­­­______years ______ months | |

| **Section 1: General information on reactive surveillance and response approaches and malaria elimination programme** | | |
| --- | --- | --- |
|  | Does your malaria programme follow (or aim to follow) a time-bound strategy for case reporting, case investigation and response activities? | 1. No 2. Yes, the 1-3-7 approach 3. Yes, another approach (please specify): |
|  | Where does this time-bound strategy for case reporting, case investigation and response activities apply? | 1. Areas in elimination phase 2. All areas 3. Other (please specify) |

| **Section 2: Case Notification** | | |
| --- | --- | --- |
|  | How are positive malaria cases initially reported? | 1. Paper based reporting 2. Electronic reporting system 3. Telephone call 4. Messaging program (e.g. WhatsApp) |
|  | In your opinion, how frequently are cases reported within 24 hours of diagnosis? | 1. Never 2. Occasionally (less than 20%) 3. Sometimes (20-50%) 4. More often than not (50-75%) 5. Usually (more than 75%) 6. Nearly always (more than 90%) |

| **Section 2: Case Investigation** | | |
| --- | --- | --- |
|  | What event triggers a case investigation? | 1. Case reported to national level 2. Case reported to peripheral level 3. Other, please specify: |
|  | What is the policy for conducting a case investigation | 1. All indigenous and imported cases 2. Indigenous cases only 3. Imported cases only 4. Other – please specify: |
|  | To your knowledge, how frequently is case investigation completed for positive malaria cases | 1. For all cases (100%) 2. For <20% of cases 3. Between 20% and 50% of cases 4. Between 50% and 75% of cases 5. More than 75% of cases but less than 100% |
|  | If all cases are not investigated, what are the main reasons these cases are not investigated? | 1. It is an imported case 2. It is outside of the district of the person investigating 3. The person could not be found 4. Not enough staff/resources 5. Daily cross-border case 6. Not applicable – every case is investigated 7. Other – please specify: |
|  | What occurs for cases that are not investigated? |  |
|  | Is there a specific person in the malaria programme who is responsible for overseeing case investigations? | 1. Yes, please specify title/role 2. No |
|  | Who is responsible for performing case investigations in your malaria programme? | 1. Village Health Volunteers or equivalent 2. Add other options |
|  | Is this person trained in case investigation techniques? | 1. Yes 2. No |
|  | If so, how frequent is the training relating to case investigations? | 1. Monthly 2. Quarterly 3. Yearly 4. Every second year |
|  | Are personnel conducting investigations periodically supervised by managers? | 1. Yes 2. No |
|  | How regularly are personnel conducting investigations supervised by managers or other superiors? | 1. Monthly 2. Quarterly 3. Yearly 4. Other – please specify |
|  | How soon after a positive case is recorded is a case investigation initiated? | 1. Within 24 hours 2. Within 48 hours 3. Within 72 hours 4. Within one week 5. Within one month |
|  | Is there an SOP for case investigation? | 1. Yes 2. No |
|  | Is there a specific case investigation form to use when conducting investigations? | 1. Yes 2. No |
|  | Does case investigation involve visiting the index case? | 1. Yes, always 2. No, never 3. Yes, sometimes |
|  | When doing a case investigation, which of the following best describes how an appointment is made with the index case (choose one) | 1. Telephone the index case 2. No prior communication – go to the index case residence to see if they are home 3. Other – please specify |
|  | What is done if the index case is not home when they visit? (select all that apply) | 1. Visit a second time: later that day or on a subsequent day 2. Telephone to schedule an appointment 3. We mark the case as imported 4. We mark the case as “not found” 5. We do not re-visit the index case 6. Inform volunteers to make appointment with the case 7. Other – Please specify: |
|  | What time of day do you normally investigate cases? (choose all that apply) | 1. Right after case detected at health facility 2. Before 8am 3. Between 8am-12noon 4. Between 12noon-5pm 5. After 5pm 6. Weekends 7. Whenever the team is available |
|  | If all cases are not investigated, what are the main reasons these cases are not investigated?  (select all that apply) | 1. It is an imported case 2. It is outside of my district 3. The person could not be found 4. Not enough staff/resources 5. Daily cross-border case 6. Not applicable – we investigate every case 7. Other – Please specify: |
|  | Is supervised treatment conducted for positive cases? | 1. Yes 2. No |
|  | Is follow-up on adherence to treatment conducted for each case? | 1. Yes 2. No |
|  | Do case investigations involve checking on malaria prevention measures used by the index case? | 1. Yes 2. No |
|  | Do case investigations involve educating the index case on malaria risk factors and prevention? | 1. Yes 2. No |
|  | What information is used to determine if an index case is imported or local? Case has travelled to another endemic area (choose all that apply) | 1. Village 2. District 3. Province/State 4. Region 5. Country 6. Any of the above 7. Other – please specify: |
|  | Do case investigations involve mapping the location of the index case and if so how is this performed? E.g. geographical information system (GIS) to make the maps. | 1. No 2. Yes, explain: |
| X | Does your programme collect information from index cases on their travel history? | 1. Yes 2. No |
|  | If yes to X, does your programme collect information on travel within the district of residence? | 1. Yes 2. No |
|  | If yes to X, does your programme collect information on travel outside the district of residence? | 1. Yes 2. No |
|  | If yes to X, does your programme collect information on travel outside of the country? | 1. Yes 2. No |
|  | How does your programme define imported cases? | 1. Cases originating in another country 2. Cases occurring within the country but from a different province, district, or other administrative unit 3. Other, specify____ |
|  | Does your programme/country collect data and report on intra-country importation of cases (e.g. from different districts) | 1. Yes 2. No |
|  | What are some of the challenges in conducting case investigation? |  |

| **Section 3: Foci Investigations and Reactive Case Detection** | | |
| --- | --- | --- |
|  | Does your programme routinely conduct foci investigations? | 1. Yes 2. No |
| Y | Does your programme routinely conduct reactive case detection? | 1. Yes 2. No |
| 3.2 | If yes to Y, what is the trigger for reactive case detection in your programme/country? | 1. Every indigenous case is a trigger (e.g. one case identified through passive case detection considered to be local) 2. All imported cases irrespective of duration of stay 3. Imported cases if they have stayed more than a certain number of days in country, specify no. days____ |
|  | How soon after a positive case is recorded is a foci investigation initiated (where necessary)? | 1. Within 24 hours 2. Within 48 hours 3. Within 72 hours 4. Within seven days 5. Within 14 days 6. Within 28 days |
|  | How soon after a positive case is investigated is a foci investigation initiated (where necessary)? | 1. Within 24 hours 2. Within 48 hours 3. Within 72 hours 4. Within seven days 5. Within 14 days 6. Within 28 days |
|  | What is the threshold number of infections, identified through passive case detection that triggers reactive case detection in your programme? |  |
|  | Do you screen household members of the index case? | 1. Always 2. Never 3. Sometimes – please explain: |
|  | When screening household members do you screen: | 1. Febrile cases only 2. All household members (asymptomatic and febrile cases) 3. We do not screen household members of a positive case |
|  | What is done if someone from the household of the index case is not home and they cannot be screened? (Check all that apply) | 1. Visit the household later that day or on a subsequent day 2. Schedule an appointment with the household members to return 3. We do not return 4. Other – Please specify: |
|  | How often do you screen neighbours of the index case in the community? | 1. Always 2. Sometimes 3. Never |
|  | When screening neighbours of the index case, are febrile individuals tested or all individuals | 1. Febrile neighbours only 2. All neighbours 3. Not applicable |
|  | What triggers screening in the community? | 1. Local cases only 2. Local and imported cases 3. Imported cases only 4. When local cases reach a minimum threshold (please specify below): |
|  | Do you screen a minimum number of households around a positive index case | 1. No 2. Yes (specify minimum number of households): |
|  | Do you screen a minimum number of people around a positive index case | 1. No 2. Yes (specify minimum number of individuals): |
|  | Do you screen within a minimum geographic radius around a positive index case | 1. No 2. Yes (specify number of meters radius screened): |
|  | What time of day do you normally conduct screening in the community? (Circle all that apply) | 1. Right after case is detected at the health facility or in the community 2. Before 8am 3. Between 8am-12noon 4. Between 12noon-5pm 5. After 5pm 6. Weekdays 7. Weekends 8. Whenever the team is available |
|  | If someone is missing at the time of screening in the community, do you return to screen them? | 1. Yes 2. No 3. If you do not return, what do you do to reach that individual? |
|  | When conducting RACD, which diagnostic method is used (mark all that apply)  are used and some are used in combination with others for diagnosis confirmation and speciation (results not mutually exclusive). All (13) respondents reported using microscopy, seven use rapid diagnostic tests (RDT), five use polymerase chain reaction (PCR), two use clinical diagnosis, and one uses serology (Table 5). | 1. Microscopy 2. Rapid diagnostic test (RDT) 3. Polymerase chain reaction (PCR) 4. Clinical diagnosis 5. Serology |
|  | What is the trigger for reactive case detection in your malaria program | 1. Single confirmed case 2. >1 confirmed case within a specified radius 3. Other threshold of confirmed cases (describe)____________________ |
|  | What are some of the challenges in conducting screening in the community? |  |

| **Section 4: Response activities** | | | | |
| --- | --- | --- | --- | --- |
|  | What kinds of response activities may be triggered when a malaria case or focus is identified? | | 1. Raising awareness about causes of malaria transmission 2. Raising awareness about malaria prevention 3. Providing additional vector control if needed 4. Entomological surveillance 5. If entomological surveillance not possible, performing spot checks for mosquito breeding grounds | |
|  | How soon after a malaria case is initially reported are response activities usually commenced? | | 1. Within 24 hours 2. Within 48 hours 3. Within 72 hours 4. Within seven days 5. Within 14 days 6. Within 28 days | |
|  | How soon after a malaria case is investigated are response activities usually commenced? | | 1. Within 24 hours 2. Within 48 hours 3. Within 72 hours 4. Within seven days 5. Within 14 days 6. Within 28 days | |
|  | How soon after a malaria foci is initially investigated are response activities usually commenced? | | 1. Within 24 hours 2. Within 48 hours 3. Within 72 hours 4. Within seven days 5. Within 14 days 6. Within 28 days | |
|  | Within your malaria program are there any reactive surveillance and response activities specifically targeted to mobile and migrant populations, including forest-goers? | | Need to refine/expand this question | |
|  | | In your experience, does information gained from case and foci investigations and classification influence the kinds of response activities that are carried out. If so, how? | |  |
|  | Do you think current reactive surveillance and response activities are sufficient for targeting P. vivax malaria? | | Need to refine/expand this question | |

| **Section 5: Facilitators and Barriers to implementation and adherence to current reactive surveillance and response strategies** | | |
| --- | --- | --- |
|  | What do you think are the current barriers (if any) to conducting case notification within 1 day? | Add options or keep as open ended?? |
|  | What do you think are the current barriers (if any) to conducting case investigations within 3 days? |  |
|  | What do you think are the current barriers (if any) to conducting foci investigation and response within 7 days? |  |
|  | What are the current barriers to following current guidelines for implementation of reactive surveillance and response activities? |  |
|  | In your experience, has the COVID-19 pandemic had an impact on successful implementation of reactive surveillance and response strategies? |  |

### Questionnaire 2 For surveying frontline malaria service providers

| **General background Information** | | |
| --- | --- | --- |
| 0.1 | Date (DD/MM/YYYY): |  |
| 0.2 | Country: |  |
| 0.3 | Name of organization: |  |
| **Demographic information on frontline malaria service provider** | | |
| 0.4 | Completed age (in years): |  |
| 0.5 | Gender | 1. Male 2. Female 3. Other/Prefer not to say |
| 0.6 | Highest education level | 1. No formal education 2. Primary school level 3. Secondary school level 4. High school level 5. Degree holder 6. Others (specify) |
| 0.7 | What is your current role? |  |
| 0.8 | How long have you worked in your current role | ­­­______years ______ months |

| **Section 1: Village/worksite characteristics** | | |
| --- | --- | --- |
| 1.1 | Approximately how many households are in your village/worksite(s) or catchment area (for health facility)? | ___ households |
| 1.2 | What is the approximate total population size of your village/worksite(s) or catchment area (for health facility)? | ___ persons |
| 1.3 | Is there mobile phone signal in your village/worksite (s)? (Any SIM card) | 1. Yes 2. No |
| 1.4 | If yes, is the mobile phone signal good at your village/worksite(s)? | 1. Yes 2. No |
| 1.5 | Is there Internet access at your village/worksite(s)? | 1. Yes 2. No |
| 1.6 | If yes, is the internet access good at your village/worksite(s)? | 1. Yes 2. No |

| **Section 2: Malaria testing and case notification** | | |
| --- | --- | --- |
| 3.1 | On average, how many malaria diagnostic tests do you perform in one month? | ­­___ tests |
| 3.2 | On average, how many days per month do you perform malaria rapid diagnostic tests? | ___ days |
| 3.3 | In general, how are you notified about possible cases of malaria in your village/worksite(s)? **(Select all that apply)** | 1. Phone call from patient 2. Phone call/referral from patient’s family member or friend 3. Patient’s visit to volunteer 4. House visit by volunteer 5. Mass testing 6. Other (Specify): |
|  | How do you first report positive malaria cases to your supervisor/organization? | 1. Paper based reporting 2. Electronic reporting system (e.g. mobile or tablet) 3. Telephone call 4. Messaging program (e.g. WhatsApp) |
|  | How frequently are you able to report malaria cases within 24 hours of diagnosis? | 1. Never 2. Occasionally (less than 20%) 3. Sometimes (20-50%) 4. More often than not (50-75%) 5. Usually (more than 75%) 6. Nearly always (more than 90%) |

| **Section 3: General information on reactive surveillance and response approaches and malaria elimination programme** | | |
| --- | --- | --- |
|  | Within the malaria programme you work for, are you aware of a time-bound strategy for case reporting, case investigation and response activities? | 1. No 2. Yes, the 1-3-7 approach 3. Yes, another approach (please specify) |
|  | Where does this time-bound strategy for case reporting, case investigation and response activities apply? | 1. Areas in elimination phase 2. All areas 3. Other (please specify) |

| **Section 4: Case Investigation** | | |
| --- | --- | --- |
|  | What is the policy for conducting a case investigation in your malaria programme and area? | 1. All indigenous and imported cases 2. Indigenous cases only 3. Imported cases only 4. Other – please specify: 5. Don’t know |
|  | What event triggers a case investigation? | 1. Case reported to national level 2. Case reported to peripheral level 3. Other, please specify: 4. Don’t know |
|  | Are you personally involved in malaria case investigations? | 1. Yes, always 2. Yes, sometimes 3. No, never |
|  | If you are involved in malaria case investigations are you supervised in these activities? | 1. Yes, by ____ 2. No |
|  | If you are not involved in case investigations, would you be willing to be involved in them in future? | 1. Yes 2. No |
|  | With respect to positive malaria cases identified by you or referred to you, how frequently is case investigation completed? | 1. For all cases (100%) 2. For <20% of cases 3. Between 20% and 50% of cases 4. Between 50% and 75% of cases 5. More than 75% of cases but less than 100% |
|  | If all cases are not investigated, what are the main reasons these cases are not investigated? | 1. It is an imported case 2. It is outside of the district of the person investigating 3. The person could not be found 4. Not enough staff/resources 5. Daily cross-border case 6. Not applicable – every case is investigated 7. Other – please specify: |
|  | What occurs for cases that are not investigated? |  |
|  | How regularly are personnel conducting investigations supervised by managers or other superiors? | 1. Monthly 2. Quarterly 3. Yearly 4. Other – please specify |
|  | How soon after a positive case is recorded is a case investigation initiated? | 1. Within 24 hours 2. Within 48 hours 3. Within 72 hours 4. Within one week 5. Within one month |
|  | Is there an SOP for case investigation? | 1. Yes 2. No |
|  | Do you follow the SOP for case investigations when required to conduct case investigations? | 1. Yes 2. No |
|  | Have you personally been trained to conduct case investigations? | 1. Yes 2. No |
|  | If so, how frequent is the training for case investigations? | 1. Monthly 2. Quarterly 3. Yearly 4. Every second year |
|  | Is there a specific case investigation form to use when conducting investigations? | 1. Yes 2. No |
|  | Does case investigation involve visiting the index case? | 1. Yes, always 2. No, never 3. Yes, sometimes |
|  | When doing a case investigation, which of the following best describes how you make an appointment with that index case? (choose one) | 1. Telephone the index case 2. No prior communication – go to the index case residence to see if they are home 3. Other – please specify |
|  | What do you do if the index case is not home when you visit? (select all that apply) | 1. Visit a second time: later that day or on a subsequent day 2. Telephone to schedule an appointment 3. We mark the case as imported 4. We mark the case as “not found” 5. We do not re-visit the index case 6. Inform volunteers to make appointment with the case 7. Other – Please specify: |
|  | What time of day do you normally investigate cases? (choose all that apply) | 1. Right after case detected at health facility 2. Before 8am 3. Between 8am-12noon 4. Between 12noon-5pm 5. After 5pm 6. Weekends 7. Whenever the team is available |
|  | If all cases are not investigated, what are the main reasons these cases are not investigated?  (select all that apply) | 1. It is an imported case 2. It is outside of my district 3. The person could not be found 4. Not enough staff/resources 5. Daily cross-border case 6. Not applicable – we investigate every case 7. Other – Please specify: |
|  | Do you conduct supervised treatment for positive cases? | 1. Always 2. Sometimes 3. Never |
|  | Do you conduct follow-up on adherence to treatment for each case? | 1. Always 2. Sometimes 3. Never |
|  | During case investigations do you check on malaria prevention measures used by the index case? |  |
|  | Do case investigations involve educating the index case on malaria risk factors and prevention? |  |
|  | What information is used to determine if an index case is imported or local? Case has travelled to another endemic area (choose all that apply) | 1. Village 2. District 3. Province/State 4. Region 5. Country 6. Any of the above 7. Other – please specify: |
|  | Do case investigations involve mapping the location of the index case and if so how is this performed? E.g. geographical information system (GIS) to make the maps. | 1. Yes 2. No |
| X | Does your programme collect information from index case on their travel history?  Nine programmes participating in the survey collect information on whether the index case has had any recent contact with travellers or immigrants. Only three countries gather patient history of glucose-6-phosphate dehydrogenase (G6PD) deficiency, an inherited blood disorder prevalent in many malaria-endemic areas. | 1. Yes 2. No |
|  | If yes to X, does your programme collect information on travel within the district of residence? , outside the district of resi | 1. Yes 2. No |
|  | If yes to X, does your programme collect information on travel outside the district of residence? | 1. Yes 2. No |
|  | If yes to X, does your programme collect information on travel outside of the country? | 1. Yes 2. No |
|  | How does your programme define imported cases? | 1. Cases originating in another country 2. Cases occurring within the country but from a different province, district, or other administrative unit 3. Other, specify____ |
|  | Does your programme/country collect data and report on intra-country importation of cases (e.g. from different districts) | 1. Yes 2. No |
|  | What are some of the challenges in conducting case investigation? |  |
|  | What role do you think malaria service providers like yourself should play in case investigation? |  |

| **Section 3: Foci Investigations and Reactive Case Detection** | | |
| --- | --- | --- |
| 3.2 | What is the trigger for reactive case detection in your programme/country? | 1. Every indigenous case is a trigger (e.g. one case identified through passive case detection considered to be local) 2. All imported cases irrespective of duration of stay 3. Imported cases if they have stayed more than a certain number of days in country, specify no. days____ |
| 3.1 | What is the threshold number of infections, identified through passive case detection that triggers reactive case detection in your programme? |  |
|  | Do you screen household members of the index case? | 1. Always 2. Never 3. Sometimes – please explain: |
| 3.3 | When screening household members do you screen: | 1. Febrile cases only 2. All household members (asymptomatic and febrile cases) 3. We do not screen household members of a positive case |
|  | What is done if someone from the household of the index case is not home and they cannot be screened? (Check all that apply) | 1. Visit the household later that day or on a subsequent day 2. Schedule an appointment with the household members to return 3. We do not return 4. Other – Please specify: |
|  | How often do you screen neighbours of the index case in the community? | 1. Always 2. Sometimes 3. Never |
|  | When screening neighbours of the index case, are febrile individuals tested or all individuals | 1. Febrile neighbours only 2. All neighbours 3. Not applicable |
|  | What triggers screening in the community? | 1. Local cases only 2. Local and imported cases 3. Imported cases only 4. When local cases reach a minimum threshold (please specify below): |
|  | Do you screen a minimum number of households around a positive index case | 1. No 2. Yes (specify minimum number of households): |
|  | Do you screen a minimum number of people around a positive index case | 1. No 2. Yes (specify minimum number of indviduals): |
|  | Do you screen within a minimum geographic radius around a positive index case | 1. No 2. Yes (specify number of meters radius screened): |
|  | What time of day do you normally conduct screening in the community? (Circle all that apply) | 1. Right after case is detected at the health facility or in the community 2. Before 8am 3. Between 8am-12noon 4. Between 12noon-5pm 5. After 5pm 6. Weekdays 7. Weekends 8. Whenever the team is available |
|  | If someone is missing at the time of screening in the community, do you return to screen them? | 1. Yes 2. No 3. If you do not return, what do you do to reach that individual? |
|  | When conducting RACD, which diagnostic method is used (mark all that apply)  are used and some are used in combination with others for diagnosis confirmation and speciation (results not mutually exclusive). All (13) respondents reported using microscopy, seven use rapid diagnostic tests (RDT), five use polymerase chain reaction (PCR), two use clinical diagnosis, and one uses serology (Table 5). | 1. Microscopy 2. Rapid diagnostic test (RDT) 3. Polymerase chain reaction (PCR) 4. Clinical diagnosis 5. Serology |
|  | To your knowledge, what is the trigger for reactive case detection in your malaria program | 1. Single confirmed case 2. >1 confirmed case within a specified radius 3. Other threshold of confirmed cases (describe)____________________ 4. Don’t know |
|  | What are some of the challenges in conducting screening in the community? |  |

| **Section 4: Response activities** | | |
| --- | --- | --- |
| 4.1 | In your role, what response activities are you involved in following identification of a malaria focus? (select all that apply) | 1. Raising awareness about causes of malaria transmission 2. Raising awareness about malaria prevention 3. Providing additional vector control if needed 4. Entomological surveillance 5. If entomological surveillance not possible, performing spot checks for mosquito breeding grounds |
|  | In your experience, does information gained from case and foci investigations and classification influence the kinds of response activities that are carried out. If so, how? |  |
|  | How soon after a malaria case is initially reported are response activities usually commenced? | 1. Within 24 hours 2. Within 48 hours 3. Within 72 hours 4. Within seven days 5. Within 14 days 6. Within 28 days |
|  | How soon after a malaria case is investigated are response activities usually commenced? | 1. Within 24 hours 2. Within 48 hours 3. Within 72 hours 4. Within seven days 5. Within 14 days 6. Within 28 days |
|  | How soon after a malaria foci is initially investigated are response activities usually commenced? | 1. Within 24 hours 2. Within 48 hours 3. Within 72 hours 4. Within seven days 5. Within 14 days 6. Within 28 days |

| **Section 5: Facilitators and Barriers to implementation and adherence to current reactive surveillance and response strategies** | | |
| --- | --- | --- |
|  | What do you think are the current barriers (if any) to conducting case notification within 1 day? | Add options or keep as open ended?? |
|  | What do you think are the current barriers (if any) to conducting case investigations within 3 days? |  |
|  | What do you think are the current barriers (if any) to conducting foci investigation and response within 7 days? |  |
|  | What are the current barriers to following current guidelines for implementation of reactive surveillance and response activities? |  |
|  | Do you have any suggestions for how current reactive surveillance and response activities can be improved? |  |
|  | In your experience, has the COVID-19 pandemic had an impact on successful implementation of reactive surveillance and response strategies |  |

### Topic guide for focus group discussion with service providers who undertake malaria case detection and case management in the field and have to directly or indirectly take part in the field level malaria RASR activities

This is the topic guide to be used by the facilitator of the focus group discussion of service provider who usually do the malaria case detection and case management in the field and have to directly or indirectly take part in the field level malaria RASR activities. It assesses their perceptions and practice of current malaria RASR activities, their opinions regarding feasibility and acceptability of these activities, and suggestions for optimization of these activities increasing their effectiveness in the current malaria elimination settings of the GMS.

This discussion is expected to be conducted in about 1.5 – 2 hours. A breaktime of 10 – 15 minutes can be incorporated into the session.

| **Person** | **Responsibility** |
| --- | --- |
| Facilitator | Lead the overall process and facilitate discussion to obtain enriched data using an ethical approach |
| Note taker | Note-taking, audio recording and supplementary facilitation |
| Translator | Translation of facilitator and participants discussion where necessary |

| **1. Information about the discussion session** | | |
| --- | --- | --- |
| 1.1. | Name of the facilitator |  |
| 1.2. | Name of the notetaker/s |  |
| 1.3. | Name of the translator/s |  |
| 1.4. | Date (dd/mm/yyyy) |  |
| 1.5. | Start time |  |
| 1.6. | End time |  |
| 1.7. | Archival code |  |

| **Is it OK to audio-record this conversion? (Yes/No)** |  |
| --- | --- |

| **2. Brief explanation of scope of the study** |
| --- |
| *“Before starting our interview, I would like to briefly explain the scope of our study. Our study focuses mainly on the malaria reactive surveillance and response (RASR) strategies and activities in the GMS countries. Malaria RASR activities are part of the overall malaria surveillance activities and they include* ***all activities to be carried once a malaria positive case has been detected by a service provider****. They can include* ***malaria case notification, case investigations, reactive case detection, focus investigation, subsequent appropriate response activities and any others****. They may sometimes be referred to as CIFIR activities in some countries.*  *So, this interview will include questions about these malaria RASR activities and other related activities.”* |

| **3. Background information of the participants** | | | | | | |
| --- | --- | --- | --- | --- | --- | --- |
| 3.1. | Could you please briefly introduce yourself, including your age, sex, designation, department, organization and your level of representativeness? | | | | | |
|  | ***Age*** | ***Sex*** | ***Designation*** | ***Department*** | ***Organization*** | ***Remark*** |
| P1 |  |  |  |  |  |  |
| P2 |  |  |  |  |  |  |
| P3 |  |  |  |  |  |  |
| P4 |  |  |  |  |  |  |
| P5 |  |  |  |  |  |  |
| P6 |  |  |  |  |  |  |
| 3.2. | How long have you been working in the current position? How long have you been working with malaria programs? | | | | | |

| **4. Existing malaria activities in the country that you need to take part in** | |
| --- | --- |
| 4.1. | Could you describe and discuss what are the malaria services currently being provided in your area??   1. What kind of malaria services are provided?  - Malaria case finding - Malaria case management (Treatment & Refer) - Malaria prevention and BCC activities - Others (if any)  1. What are the standard guidelines and procedures for implementing these malaria service provision activities? 2. Do you think these malaria service provision activities could be carried out according to the standard guidelines and procedures? Why? How could they be improved? 3. What are your roles and responsibilities in RASR activities?  - Case notification - Case investigation - Reactive case detection - Focus investigation - Response activities - Others (if any)  1. What are the standard guidelines and procedures for implementing these RASR activities? 2. What is the standard/targeted time schedule for implementing these RASR activities? *(e.g., China’s 1-3-7 strategy)* |
| 4.2. | Do you think each and every activity could be carried out according to the standard guidelines and procedures in your areas? Why?   - Case notification - Case investigation - Reactive case detection - Focus investigation - Response activities - Others (if any)  1. How about in terms of timeliness? Why? 2. How about in terms of completeness? Why? |

| **5. Feasibility of current malaria RASR strategies and activities** | |
| --- | --- |
| 5.1. | What are the challenges for strictly following the standard procedures and guidelines in implementation of these activities in your areas?   - Case notification - Case investigation - Reactive case detection - Focus investigation - Response activities - Others (if any)  1. What are the challenges in implementing RASR activities from aspects of service providers?   In terms of:   - Human resources - Commodity resources (example: RDT, Phone, Motorcycle) - Technical capacity (example: Mobile phone literacy for reporting, Difficulty in implementing RASR activities) - Communication (Phone reporting) - Transportation - Financial resources - Political situations - Cultural background   How can these challenges be overcome? What kinds of support will be needed?   1. What are the challenges for implementing RASR activities from the aspect of villagers/beneficiaries? |
| 5.2. | Do you think the time schedule of current malaria RASR activities is appropriate to be strictly followed in your current settings?   1. What are the challenges? 2. How can they be improved? What kinds of support will be needed? |
| 5.3. | What do you think are the strengths of current malaria RASR activities (probably over others)? Why?  What are the external factors favouring your current RASR activities? Why?  ***(The same probing questions as above can be used.)*** |
| 5.4. | Do you think the malaria RASR activities currently implemented in your area is a good one in terms of feasibility? Why? How can it be improved? |

| **6. Acceptability of current malaria RASR strategies and activities** | |
| --- | --- |
| 6.1. | Do you think the current malaria RASR activities are acceptable/ satisfactory for different malaria program stakeholders at different levels of implementation in different geographical areas? Why?   - Case finding and management - Case notification - Case investigation - Reactive case detection - Focus investigation - Response activities - Others (if any)   At   - Community level like yours (example: CHW/Volunteers) - Managerial level like your supervisors - Beneficiary level like the villagers including the MMPs  1. How can they be improved to make them more acceptable for the different stakeholders? |

| **7. Perceived effectiveness of current strategies** | |
| --- | --- |
| 7.1. | Do you think the current malaria RASR activities are effective for achieving malaria elimination goals in your areas? And efficient? Why? |
| 7.2. | What do you think about the malaria incidence and prevalence in your areas going in the past years?   1. Can you relate these changes to your current malaria RASR activities? How? |
| 7.3. | How do you think your current malaria RASR activities can be optimized to improve their effectiveness and efficiencies in your malaria elimination setting? What kinds of support will be needed? |

***(Skip section 8 if all participants are CHWs)***

| **8. Role of CHW in malaria RASR Strategies** | |
| --- | --- |
| 8.1. | What kind of community health workers are working with malaria elimination program in your area? What are their regular malaria elimination activities? |
| 8.2. | What are the roles of these community health workers (CHWs) in the current malaria RASR activities? How are they taking part in the different current malaria RASR activities?   - Case finding and management - Case notification - Case investigation - Reactive case detection - Focus investigation - Response activities - Any others |
| 8.3. | What are the challenges regarding the CHWs taking part in the malaria RASR activities? |
| 8.4. | How can the participation of CHWs in malaria RASR activities be optimized? (How can roles of CHWs in malaria RASR activities be optimized?) What kinds of support will be needed? |

| **9. Optimization of malaria RASR strategies for MMPs** | |
| --- | --- |
| 9.1. | What do you think about the acceptability of your current malaria RASR activities from the perspectives of the beneficiaries in your area such as the community members or the villagers? Why? |
| 9.2. | Do you have any mobile and migrant populations in your area?   1. What kind of MMPs are there? Migrant workers? IDP? 2. Immigrant? Outmigrants? 3. How many of them are there? 4. What are the seasonal dynamics of the MMPs? |
| 9.3. | What are the challenges in implementing these malaria RASR activities among the MMPs in your areas? Why? |
| 9.4. | How can the malaria RASR activities be optimized for the MMPs in your areas? What kinds of support will be needed? |

| **10. Overall** | |
| --- | --- |
| 10.1. | *On consideration of all these factors that we have discussed before,* do you think the current malaria RASR activities are effective and efficient for achieving the malaria elimination goals in your areas and in your country? Why? |
| 10.2. | *On consideration of all these factors that we have discussed before*, do you think the current malaria RASR activities are best suited with your country’s contextual background? Why? |
| 10.3. | How do you think the current malaria RASR activities can be optimized, overcoming the existing barriers and improving their effectiveness in existing malaria elimination setting in your areas and in your country? |

| **Conclusion** | |
| --- | --- |
|  | This is the end of our discussion.  Do you have any questions for me?  Thank you very much for your participation. |

| **End of session** |
| --- |

### Topic guide for focus group discussion with malaria program stakeholders responsible for managing or supervising field RASR activities

This is the topic guide to be used by the facilitator of the focus group discussion of malaria program stakeholders responsible for managing, coordinating and supervising the field level malaria RASR activities. It assesses their perceptions and practice of current malaria RASR policies and strategies, their opinions regarding feasibility and acceptability of these strategies, and suggestions for optimization of these strategies increasing their effectiveness in the current malaria elimination settings of the GMS.

This discussion is expected to be conducted in about 1.5 – 2 hours. A breaktime of 10 – 15 minutes can be incorporated into the session.

| **Person** | **Responsibility** |
| --- | --- |
| Facilitator | Lead the overall process and facilitate discussion to obtain enriched data using an ethical approach |
| Note taker | Note-taking, audio recording and supplementary facilitation |
| Translator | Translation of facilitator and participants discussion where necessary |

| **1. Information about the discussion session** | | |
| --- | --- | --- |
| 1.1. | Name of the facilitator |  |
| 1.2. | Name of the notetaker/s |  |
| 1.3. | Name of the translator/s |  |
| 1.4. | Date (dd/mm/yyyy) |  |
| 1.5. | Start time |  |
| 1.6. | End time |  |
| 1.7. | Archival code |  |

| **Is it OK to audio-record this conversion? (Yes/No)** |  |
| --- | --- |

| **2. Brief explanation of scope of the study** |
| --- |
| *“Before starting our discussion, I would like to briefly explain the scope of our study. Our study focuses mainly on the malaria reactive surveillance and response (RASR) strategies and activities in the GMS countries. Malaria RASR activities are part of the overall malaria surveillance activities and they include* ***all activities to be carried once a malaria positive case has been detected by a service provider****. They can include* ***malaria case notification, case investigations, reactive case detection, focus investigation, subsequent appropriate response activities and any others****. They may sometimes be referred to as CIFIR activities in some countries.*  *So, this interview will include questions about these malaria RASR activities and other related activities.”* |

| **3. Background information of the participants** | | | | | | |
| --- | --- | --- | --- | --- | --- | --- |
| 3.1. | Could you please briefly introduce yourself, including your age, sex, designation, department, organization and your level of representativeness? | | | | | |
|  | ***Age*** | ***Sex*** | ***Designation*** | ***Department*** | ***Organization*** | ***Remark*** |
| P1 |  |  |  |  |  |  |
| P2 |  |  |  |  |  |  |
| P3 |  |  |  |  |  |  |
| P4 |  |  |  |  |  |  |
| P5 |  |  |  |  |  |  |
| P6 |  |  |  |  |  |  |
| 3.2. | How long have you been working in the current position? How long have you been working with malaria programs? | | | | | |
| 3.3. | What are your roles and responsibilities relating the malaria elimination program (especially the malaria RASR activities)? | | | | | |

| **4. Existing malaria RASR strategies in the country and current practice** | |
| --- | --- |
| 4.1. | Could you describe and discuss what are the malaria RASR policies and strategies currently being implemented in your areas?   1. What kind of activities are included?  - Case notification - Case investigation - Reactive case detection - Focus investigation - Response activities - Others (if any)  1. What are the standard guidelines and procedures for implementing these activities? 2. What is the standard/targeted time schedule for implementing these activities? *(e.g., China’s 1-3-7 strategy)* |
| 4.2. | Do you think each and every activity of your strategies could be carried out according to your standard guidelines and procedures in your areas? Why?   - Case notification - Case investigation - Reactive case detection - Focus investigation - Response activities - Others (if any)  1. How about in terms of timeliness? Why? 2. How about in terms of completeness? Why? |
| 4.3. | Does anyone of you have to give services for malaria case finding and case management in the field?   1. Do you think malaria case finding and case management could be carried out according to your standard guidelines and procedures? Why? 2. How could they be improved? |

| **5. Feasibility of current malaria RASR strategies and activities** | |
| --- | --- |
| 5.1. | What are the challenges for strictly following the standard procedures and guidelines in implementation of these activities in your areas?   - Case notification - Case investigation - Reactive case detection - Focus investigation - Response activities - Others (if any)  1. What are the health system-related challenges?   In terms of:   - Political commitments - Human resources - Financial resources - Commodity resources - Technology - Any others   How can these challenges be overcome? What kinds of support will be needed?   1. What are the challenges related to existing background infrastructure of your areas?   In terms of:   - Communication - Transportation - Political situation - Cultural background - Any others   How can these challenges be overcome? What kinds of support will be needed?   1. Has the COVID-19 pandemic affected how RASR strategies and activities are carried out in your area? If so, how? |
| 5.2. | Do you think the time schedule of your current malaria RASR strategies is appropriate to be strictly followed in your current settings?   1. What are the challenges? 2. How can they be improved? What kinds of support will be needed? |
| 5.3. | What do you think are the strengths of your current malaria RASR strategies (probably over others)? Why?  What are the external factors favouring your current RASR strategies? Why?  ***(The same probing questions as above can be used.)*** |
| 5.4. | Do you think the malaria RASR strategies currently implemented in your area is a good one in terms of feasibility? Why? How can it be improved? |

| **6. Acceptability of current malaria RASR strategies and activities** | |
| --- | --- |
| 6.1. | Do you think the current malaria RASR strategies and its activities are acceptable/ satisfactory for different malaria program stakeholders at different levels of implementation in different geographical areas? Why?   - Case notification - Case investigation - Reactive case detection - Focus investigation - Response activities - Others (if any)   At   - Field implementation level like yours - Managerial level like your supervisors - Community level like the CHWs - Beneficiary level like the villagers including the MMPs  1. How can they be improved to make them more acceptable for the different stakeholders? |

| **7. Perceived effectiveness of current strategies** | |
| --- | --- |
| 7.1. | Do you think the current malaria RASR strategies are effective for achieving malaria elimination goals in your areas? And efficient? Why? |
| 7.2. | What do you think about the malaria incidence and prevalence in your areas going in the past years?   1. Can you relate these changes to your current malaria RASR strategies? How? |
|  | Is information gained from case and foci investigations currently used for decision making around response activities? If so, how? |
| 7.3. | How do you think your current malaria RASR strategies can be optimized to improve their effectiveness and efficiencies in your malaria elimination setting? What kinds of support will be needed? |

| **8. Role of CHW in malaria RASR Strategies** | |
| --- | --- |
| 8.1. | What kind of community health workers are working with malaria elimination program in your area? What are their regular malaria elimination activities? |
| 8.2. | What are the roles of these community health workers (CHWs) in the current malaria RASR strategies? How are they taking part in the different current malaria RASR activities?   - Case notification - Case investigation - Reactive case detection - Focus investigation - Response activities - Any others |
| 8.3. | What are the challenges regarding the CHWs taking part in the malaria RASR activities? |
| 8.4. | How can the participation of CHWs in malaria RASR activities be optimized? (How can roles of CHWs in malaria RASR strategies be optimized?) What kinds of support will be needed? |

| **9. Optimization of malaria RASR strategies for MMPs** | |
| --- | --- |
| 9.1. | What do you think about the acceptability of your current malaria RASR strategies and activities from the perspectives of the beneficiaries in your area such as the community members or the villagers? Why? |
| 9.2. | Do you have any mobile and migrant populations in your area?   1. What kind of MMPs are there? Migrant workers? IDP? 2. Immigrant? Outmigrants? 3. How many of them are there? 4. What are the seasonal dynamics of the MMPs? |
| 9.3. | What are the challenges in implementing these malaria RASR activities among the MMPs in your areas? Why? |
| 9.4. | How can the malaria RASR strategies and activities be optimized for the MMPs in your areas? What kinds of support will be needed? |

| **10. Overall** | |
| --- | --- |
| 10.1. | *On consideration of all these factors that we have discussed before,* do you think the current malaria RASR strategies are effective and efficient for achieving the malaria elimination goals in your areas and in your country? Why? |
| 10.2. | *On consideration of all these factors that we have discussed before*, do you think the current malaria RASR strategies are best suited with your existing health system, infrastructural and socio-cultural backgrounds of your areas? Why? |
| 10.3. | How do you think the current malaria RASR strategies can be optimized, overcoming the existing barriers and improving their effectiveness in existing malaria elimination setting in your areas and in your country? |

| **Conclusion** | |
| --- | --- |
|  | This is the end of our discussion.  Do you have any questions for me?  Thank you very much for your participation. |

| **End of session** |
| --- |

### Topic guide for focus group discussion with mobile and migrant populations

This is the topic guide to be used by the facilitator of the focus group discussion of mobile and migrants who are at risks of contracting malaria. It assesses their perceptions and practice of current malaria RASR activities, their opinions regarding feasibility and acceptability of these activities, and suggestions for optimization of these activities increasing their effectiveness in the current malaria elimination settings of the GMS.

This discussion is expected to be conducted in about 1 – 1.5 hours. A breaktime of 10 – 15 minutes can be taken in between the session.

| **Person** | **Responsibility** |
| --- | --- |
| Facilitator | Lead the overall process and facilitate discussion to obtain enriched data using an ethical approach |
| Note taker | Note-taking, audio recording and supplementary facilitation |
| Translator | Translation of facilitator and participants discussion where necessary |

| **1. Information about the discussion session** | | |
| --- | --- | --- |
| 1.1. | Name of the facilitator |  |
| 1.2. | Name of the notetaker/s |  |
| 1.3. | Name of the translator/s |  |
| 1.4. | Date (dd/mm/yyyy) |  |
| 1.5. | Start time |  |
| 1.6. | End time |  |
| 1.7. | Archival code |  |

| **Is it OK to audio-record this conversion? (Yes/No)** |  |
| --- | --- |

| **2. Brief explanation of scope of the study** |
| --- |
| *“Before starting our interview, I would like to briefly explain the scope of our study. Our study focuses mainly on the malaria reactive surveillance and response (RASR) strategies and activities in the GMS countries. Malaria RASR activities are part of the overall malaria surveillance activities and they include* ***all activities to be carried once a malaria positive case has been detected by a service provider****. They can include* ***malaria case notification, case investigations, reactive case detection, focus investigation, subsequent appropriate response activities and any others****. They may sometimes be referred to as CIFIR activities in some countries.*  *So, this interview will include questions about these malaria RASR activities and other related activities.”* |

| **3. Background information of the participants** | | | | | |
| --- | --- | --- | --- | --- | --- |
| 3.1. | Could you please briefly introduce yourself, including your age, sex, village/ workplace, type and nature of migrant works? | | | | |
|  | ***Age*** | ***Sex*** | ***Village/ Worksite/ Camp*** | ***Type of MMP (To be noted by facilitator)*** | ***Remark*** |
| P1 |  |  |  |  |  |
| P2 |  |  |  |  |  |
| P3 |  |  |  |  |  |
| P4 |  |  |  |  |  |
| P5 |  |  |  |  |  |
| P6 |  |  |  |  |  |
| 3.2. | How long have you been working/ living in this village/ worksite/ camp? | | | | |
| 3.3. | Could you please describe your work nature?   1. What kinds of work do you do? 2. Immigrant? Outmigrant? 3. How many people like you in your village/ worksite/ camp? 4. What are the seasonal dynamics of the MMPs? | | | | |

| **4. Perception and experience on malaria and RASR activities** | |
| --- | --- |
| 4.1. | Could you please briefly describe current malaria situation in your area? (Common village places and MMP’s workplace)   1. Could you please describe and discuss what kinds of service provider are providing malaria service in your area? 2. What are the malaria services currently being received in your area?  - Malaria testing - Malaria case management (Treatment & Refer) - Malaria prevention and BCC services - Others (if any)  1. Could you please describe your experience in receiving malaria case management and outcomes as a malaria suspect or patient or family and friend or neighbours or co-workers? |
| 4.2. | Could you please describe what kinds of activity are carried on by the service provider when a malaria positive patient has been detected, apart from malaria testing, case management and prevention and BCC activities?   - Case notification - Case investigation - Reactive case detection - Focus investigation - Response activities   How are these activities conducted by the service provider? (Mechanism of implementing these activities)   - - Common village places   - MMP’s workplaces |

| **Brief explanation of the mechanisms of current malaria RASR activities in the area investigated by the facilitator.**  *“As explain earlier, malaria reactive surveillance and response (RASR) activities* ***are to be carried out once a malaria positive case has been detected by a service provider****. These activities can include* ***malaria case notification (mechanism), case investigations (mechanism), reactive case detection (mechanism), focus investigation (mechanism), subsequent appropriate response activities and any others****.* |
| --- |

| **5. Acceptability and Feasibility of current malaria RASR strategies and activities** | |
| --- | --- |
| 5.1. | Could you please describe how do you receive malaria testing and treatment services from the service provider if you suspect malaria?   - Do you think you are acceptable/ satisfactory in receiving these services from the service provider? Why?   Does the service provider pay a schedule visit for malaria testing and treatment services to your workplace and if yes, how frequent?   - Do you think the current service provider’s scheduled visit for malaria testing and treatment service meets your needs? Why? |
| 5.2. | Could you please describe how you cooperate in malaria RASR activities?   1. What are the challenges as MMPs in cooperating the case notification activity?  - Any sensitive issues - Others (if any)  1. What are the challenges as MMPs in cooperating the case investigation activity?  - Any sensitive issues or questions - Recalling events - Others (if any)  1. What are the challenges as MMP in cooperating the reactive case detection (RACD) activity?  - Any missing co-workers - Security issues - Others (if any)  1. What are the challenges as MMPs in cooperating the focus investigation activity? 2. What are the challenges as MMPs in cooperating the response activities?  - IRS - BCC - LLIN - Larva source management - Others (if any)   How can these challenges be overcome? What kinds of support will be needed? |
| 5.3. | What are the challenges in cooperating the RASR activities from the aspects of MMPs?  In terms of:   - Human resources - Communication (Language, mobile phone) - Transportation - Financial resources - Political situations - Cultural background - Cooperation opportunities - Others (if any)   How can these challenges be overcome? What kinds of support will be needed? |
| 5.4. | Do you think the time schedule of current malaria RASR activities is appropriate in your current settings?   1. What are the challenges? 2. How can they be improved? What kinds of support will be needed? |
| 5.5. | Do you think the malaria RASR activities currently implemented in your area is a good one in terms of feasibility? Why? How can it be improved? |

| **6. Overall** | |
| --- | --- |
| 10.1. | *On consideration of all these factors that we have discussed before,* do you think the current malaria RASR activities are acceptable/ convenient for you? Why? |
| 10.2. | *On consideration of all these factors that we have discussed before*, do you think the current malaria RASR activities are best suited with your country’s contextual background? Why? |
| 10.3. | How do you think the current malaria RASR activities can be optimized, overcoming the existing barriers and improving their effectiveness in current setting? |

| **Conclusion** | |
| --- | --- |
|  | This is the end of our discussion.  Do you have any questions for me?  Thank you very much for your participation. |

| **End of session** |
| --- |

### Topic guide for semi-structured interview with malaria program stakeholders responsible for designing and overviewing malaria reactive surveillance and response policies and strategies

This is the interview topic guide for the in-depth interview with the malaria program stakeholders responsible for designing and overviewing malaria reactive surveillance and response (RASR) policies and strategies. It assesses their perceptions and practice of current malaria RASR policies and strategies, their opinions regarding feasibility and acceptability of these strategies, and suggestions for optimization of these strategies increasing their effectiveness in the current malaria elimination settings of the GMS.

This interview is expected to be conducted in about 1 – 1.5 hours. A breaktime of 10 – 15 minutes can be incorporated into the session.

| **Person** | **Responsibility** |
| --- | --- |
| Interviewer | Lead the interview and facilitate discussion to obtain enriched data using an ethical approach |
| Note taker | Note-taking, audio recording and supplementary facilitation |
| Translator | Translation of facilitator and participants discussion where necessary |

| **Information about the interview session** | | |
| --- | --- | --- |
| 1.1. | Name of the interviewer |  |
| 1.2. | Name of the note taker |  |
| 1.3. | Date (dd/mm/yyyy) |  |
| 1.4. | Start time |  |
| 1.5. | End time |  |
| 1.6. | Archival code |  |

| **Is it OK to audio-record this conversion? (Yes/No)** |  |
| --- | --- |

| **2. Brief explanation of scope of the study** | |
| --- | --- |
| *“Before starting our interview, I would like to briefly explain the scope of our study. Our study focuses mainly on the malaria reactive surveillance and response (RASR) strategies and activities in the GMS countries. Malaria RASR activities are part of the overall malaria surveillance activities and they include* ***all activities to be carried once a malaria positive case has been detected by a service provider****. They can include* ***malaria case notification, case investigations, reactive case detection, focus investigation, subsequent appropriate response activities and any others****. They may sometimes be referred to as CIFIR activities in some countries.*  *So, this interview will include questions about these malaria RASR activities and other related activities.”* | |
| **3. Background information of the participant** | |
| 3.1. | Gender of the participant ***(Just to be noted by the interviewer)*** |
| 3.2. | Could you briefly describe your organization and your department including its coverage/catchment area? |
| 3.3. | Could you briefly describe your current designation, including its level of representativeness? |
| 3.4. | How long have you been working in the current position? How long have you been working with malaria programs? Do you mind saying your completed years of age by now? |
| 3.5. | What are your roles and responsibilities relating the malaria elimination program, especially the malaria RASR activities? |

| **4. Existing malaria RASR strategies in the country and current practice** | |
| --- | --- |
| 4.1. | Could you describe and discuss the malaria RASR policy/policies and strategy/ strategies currently being implemented in your country?   1. What kind of activities are included?  - Case notification - Case investigation - Reactive case detection - Focus investigation - Response activities - Others (if any)  1. What are the standard guidelines and procedures for implementing these activities? 2. What is the standard/targeted time schedule for implementing these activities? *(e.g., China’s 1-3-7 strategy)* 3. What are the authoritative bodies for setting these policies and strategies in your countries? 4. Do you think the strategies in your country are different from those of your neighbouring countries, especially the GMS countries? How are they different? Why? |
| 4.2. | Do you think each and every activity of your strategies could be carried out according to your standard guidelines and procedures? Why?   - Case notification - Case investigation - Reactive case detection - Focus investigation - Response activities - Others (if any)  1. How about in terms of timeliness? Why? 2. How about in terms of completeness? Why? |

| **5. Feasibility of current malaria RASR strategies and activities** | |
| --- | --- |
| 5.1. | What are the challenges for strictly following the standard procedures and guidelines in implementation of these activities?   - Case notification - Case investigation - Reactive case detection - Focus investigation - Response activities - Others (if any)  1. What are the health system-related challenges?   In terms of:   - Political commitments - Human resources - Financial resources - Commodity resources - Technology - Any others   How can these challenges be overcome? What kinds of support will be needed?   1. What are the challenges related to existing infrastructure and sociocultural background of your country?   In terms of:   - Communication - Transportation - Political situation - Cultural background - Any others   How can these challenges be overcome? What kinds of support will be needed? |
| 5.2. | Do you think the time schedule of your current malaria RASR strategies is appropriate to be strictly followed in your current settings?   1. What are the challenges? 2. How can they be improved? What kinds of support will be needed? |
| 5.3. | What do you think are the strengths of your current malaria RASR strategies (probably over others)? Why?   1. What are the external factors favouring your current RASR strategies? Why?   ***(The same probing questions as above can be used.)*** |
| 5.4. | Do you think the malaria RASR strategies currently implemented in your country is a good one in terms of feasibility? Why? How can it be improved? |
| 5.5 | Has the COVID-19 pandemic had an impact on the ability to implement malaria RASR strategies in your country? If so, how? How can this impact be mitigated? |

| **6. Acceptability of current malaria RASR strategies and activities** | |
| --- | --- |
| 6.1. | Do you think the current malaria RASR strategies and activities are acceptable/ satisfactory for different malaria program stakeholders at different levels of implementation in different geographical areas? Why?   - Case notification - Case investigation - Reactive case detection - Focus investigation - Response activities - Others (if any)   At   - Current regional and national elimination settings - Policy making & strategic level - Managing level - Field implementation level - Beneficiary level - Any others  1. How can they be improved to make them more acceptable for the different stakeholders? |

| **7. Perceived effectiveness of current strategies** | |
| --- | --- |
| 7.1. | Do you think the current malaria RASR strategies are effective for your national and regional malaria elimination goals? And efficient? Why? |
| 7.2. | What do you think about the malaria incidence and prevalence in your country going in the past years?   1. Can you relate these changes to your current malaria RASR strategies? How? |
| 7.3. | How do you think your current malaria RASR strategies can be optimized to improve their effectiveness and efficiencies in your malaria elimination setting? What kinds of support will be needed? |
| 7.4 | How is the information collected during malaria case and foci investigations used for decision making in your malaria elimination settings? Are there ways that this information could be better utilised? |

| **8. Role of CHW in malaria RASR Strategies** | |
| --- | --- |
| 8.1. | What kind of community health workers are working with malaria elimination program in your countries? What are their regular malaria elimination activities? |
| 8.2. | What are the roles of these community health workers (CHWs) in the current malaria RASR strategies? How are they taking part in the different current malaria RASR activities?   - Case notification - Case investigation - Reactive case detection - Focus investigation - Response activities - Others (if any) |
| 8.3. | What are the challenges regarding the CHWs taking part in their malaria RASR activities? |
| 8.4. | How can the participation of CHWs in malaria RASR activities be optimized? (How can roles of CHWs in malaria RASR strategies be optimized?) What kinds of support will be needed? |

| **9. Optimization of malaria RASR strategies for MMPs** | |
| --- | --- |
| 9.1. | What do you think about the acceptability of your current malaria RASR strategies and activities from the perspectives of the beneficiaries in your country such as the community members or the villagers? Why? |
| 9.2. | Do you have any mobile and migrant populations in your country?   1. What kind of MMPs are there? Migrant workers? IDP? 2. Immigrant? Outmigrants? 3. How many of them are there? 4. What are the seasonal dynamics of the MMPs? |
| 9.3. | What are the challenges in implementing these malaria RASR activities among the MMPs? Why? |
| 9.4. | How can the malaria RASR strategies and activities be optimized for the MMPs? What kinds of support will be needed? |

| **10. Overall** | |
| --- | --- |
| 10.1. | On consideration of all these factors that we have discussed before, do you think the current malaria RASR strategies in your country are effective and efficient for achieving the national and regional malaria elimination goals? Why? |
| 10.2. | On consideration of all these factors that we have discussed before, do you think the current malaria RASR strategies in your country are best suited with your existing health system, infrastructural and socio-cultural backgrounds? Why? |
| 10.3. | How do you think the current malaria RASR strategies can be optimized, overcoming the existing barriers and improving their effectiveness in existing malaria elimination setting in your country? And in the GMS? |

| **Conclusion** | |
| --- | --- |
|  | This is the end of our interview.  Do you have any questions for me?  Thank you very much for your participation. |

| **End of session** |
| --- |
