# Supplementary material for: Evaluating malaria reactive surveillance and response strategies in northeast Cambodia: a mixed-methods study
Source: Malar J. 2025 Jul 13;24:229. doi: 10.1186/s12936-025-05475-7 (PMC12255966; doi:10.1186/s12936-025-05475-7)
Supplement: Supplementary file 3 — Additional file 3. Supplementary tables [file 12936_2025_5475_MOESM3_ESM.docx]

# Additional file 3: Supplementary tables

## Supplementary table 1: Background information of the survey participants

| Variables |  | **Malaria program stakeholders**  n =40 | **Frontline service provider**  n =40 | **Total**  **N =80** |
| --- | --- | --- | --- | --- |
|  |  | n (%) | n (%) | n (%) |
| **Completed age (in years)** | | | | |
| Median (IQR) | | 36.5 (33.8 to 49.8) | 37.5 (32.0 to 43.5) | 37.0 (32.0 to 45.5) |
| **Gender** | | | | |
| Female | | 3 (7.5) | 6 (15.0) | 9 (11.2) |
| Male | | 37 (92.5) | 34 (85.0) | 71 (88.8) |
| **Highest education level** | | | | |
| Degree holder | | 18 (45.0) |  | 18 (22.5) |
| High school level | | 18 (45.0) | 3 (7.5) | 21 (26.2) |
| Primary school level | | 1 (2.5) | 19 (47.5) | 20 (25.0) |
| Secondary school level | | 3 (7.5) | 5 (12.5) | 8 (10.0) |
| Others | |  | 13 (32.5) | 13 (16.2) |
| Read and write | |  | 11 (84.6) | 11 (13.8) |
| **Current role** | | | | |
| Health centre malaria stakeholder | | 11 (27.5) |  | 11 (13.8) |
| Health centre malaria supervisor | | 25 (62.5) |  | 25 (31.2) |
| Operation district malaria supervisor | | 2 (5.0) |  | 2 (2.5) |
| Provincial Malaria Assistant | | 1 (2.5) |  | 1 (1.2) |
| Provincial Malaria stakeholder | | 1 (2.5) |  | 1 (1.2) |
| Mobile malaria worker | |  | 3 (7.5) | 3 (3.8) |
| Village malaria worker | |  | 37 (92.5) | 37 (46.2) |
| **Duration of working in current role (year)** | | | | |
| Median (IQR) | | 7.6 (4.8 to 11.0) | 7.5 (3.0 to 10.2) | 7.6 (3.2 to 10.7) |

## **Supplementary table 2: Participants of focus group discussion and in-depth interview**

|  | **Female** | **Male** | **Total** |
| --- | --- | --- | --- |
| **Focus Group Discussion** |  |  |  |
| Malaria supervisor | 2 | 16 | 18 |
| Village Malaria Worker | 1 | 18 | 19 |
| Mobile and migrant people | 8 | 14 | 22 |
| **Total** | **11** | **48** | **59** |
| **In-depth Interview** |  |  |  |
| Operational district malaria stakeholder | 1 | 2 | 3 |
| Senior Monitoring & Evaluation officer |  | 1 | 1 |
| Senior Village Malaria Worker officer |  | 1 | 1 |
| **Total** | **1** | **4** | **5** |

## Supplementary table 3: Awareness on the reactive surveillance and response strategy (survey)

|  |  | **Malaria program stakeholders**  **(n=40)** | **Frontline health workers**  **(n=40)** | **Total**  **(N=80)** |
| --- | --- | --- | --- | --- |
|  | | n (%) | n (%) | n (%) |
| **Does your program follow a time-bound strategy?** | | | | |
| Yes, 1-3-7 approach  Yes, another approach | | 39 (97.5) | 39 (97.5) | 78 (97.5) |
|  |  | 1 (2.5) | 1 (2.5) | 2 (2.5) |
| **Where does this time-bound strategy is applied for reactive surveillance and response activities?** | | | | |
| All areas | | 20 (50.0) | 13 (32.5) | 33 (41.2) |
| Areas in elimination phase | | 20 (50.0) | 27 (67.5) | 47 (58.8) |

## Supplementary table 4: Setting of the villages of frontline health workers (survey)

| **Approximately how many households are in your village/worksite(s) or catchment area (for health facility)?** | **N=40** |
| --- | --- |
| Median (IQR) | 1402 (1117 to 3004) |
| **What is the approximate total population size of your village/worksite(s) or catchment area (for health facility)?** | |
| Median (IQR) | 7364 (4973 to 10000) |
|  | **n (%)** |
| **Is there mobile phone signal in your village/worksite (s)?** | |
| Yes | 39 (97.5) |
| No | 1 (2.5) |
| **If yes, is the mobile phone signal good at your village/worksite(s)?** | |
| Yes | 26 (66.7) |
| No | 13(33.3) |
| **Is there Internet access at your village/worksite(s)?** | |
| Yes | 38 (95.0) |
| No | 2 (5.0) |
| **If yes, is the internet access good enough to do reporting at your village/worksite(s)?** | |
| Yes | 18 (47.4) |
| No | 20 (52.6) |

## Supplementary table 5: Methods of case notification (survey)

|  |  | **Malaria program stakeholders**  **(n=40)** | **Frontline health workers**  **(n=40)** | **Total**  **(N=80)** |
| --- | --- | --- | --- | --- |
|  | | n (%) | n (%) | n (%) |
| **How are positive malaria cases initially reported?** | | | | |
| Telephone calling | | 24 (60.0) | 34 (85.0) | 58 (72.5) |
| Electronic reporting system | | 32 (80.0) | 24 (60.0) | 56 (70.0) |
| Messaging applications such as WhatsApp | | 9 (22.5) | 4 (10.0) | 13 (16.2) |
| Paper-based reporting | | 7 (17.5) | 3 (7.5) | 10 (12.5) |

## Supplementary table 6: Reasons for not completing case investigation (survey)

|  | **Malaria program stakeholders**  **(n=40)** | | **Frontline health workers**  **(n=40)** | | **Total**  **(N=80)** | |  |
| --- | --- | --- | --- | --- | --- | --- | --- |
|  | n (%) | | n (%) | | n (%) | |  |
| It is an imported case | | 8 (20.0) | | 6 (15.0) | | 14 (17.5) | |
| It is outside of the district of the person investigating | | 10 (25.0) | | 12 (30.0) | | 22 (27.5) | |
| The person could not be found | | 5 (12.5) | | 4 (10.0) | | 9 (11.2) | |
| Not enough staff/resources | | 2 (5.0) | | 1 (2.5) | | 3 (3.8) | |
| Daily cross-border case | | 2 (5.0) | | 3 (7.5) | | 5 (6.2) | |
| Not applicable - every case is investigated | | 13 (32.5) | | 14 (35.0) | | 27 (33.8) | |

## Supplementary table 7: Initiating case investigation (survey)

|  | **Malaria program stakeholders**  **(n=40)** | **Frontline health workers**  **(n=40)** | **Total**  **(N=80)** |  |
| --- | --- | --- | --- | --- |
|  | *n (%)* | *n (%)* | *n (%)* |  |
| **What event triggers a case investigation?** | | | |  |
| Case reported to national level | 23 (57.5) | 10 (25.0) | 33 (41.2) |  |
| Case reported to peripheral level | 25 (62.5) | 29 (72.5) | 54 (67.5) |  |
| Others | 2 (5.0) | 3 (7.5) | 5 (6.2) |  |
| Don’t know | 0 (0) | 2 (5.0) | 2 (5.0) |  |
| **What is the policy for conducting case investigation?** | | | |  |
| All indigenous and imported cases | 26 (65.0) | 30 (75.0) | 56 (70.0) |  |
| Indigenous cases only | 11 (27.5) | 6 (15.0) | 17 (21.2) |  |
| Imported cases only | 2 (5.0) | 2 (5.0) | 4 (5.0) |  |
| Others | 1 (2.5) | 2 (5.0) | 3 (3.8) |  |
| **When doing a case investigation, which of the following best describes how an appointment is made with the index case?** | | | |  |
| Telephone the index case | 29 (72.5) | 23 (57.5) | 52 (65.0) |  |
| Visit the index case residence to see if they are home | 10 (25.0) | 17 (42.5) | 27 (33.8) |  |
| Others | 1 (2.5) | 0 (0.0) | 1 (1.2) |  |
| **What is done if the index case is not home when visited?** | | | | |
| Telephone to schedule an appointment | | 28 (70.0) | 33 (82.5) | 61 (76.2) |
| Visit second time later that day | | 25 (62.5) | 21 (52.5) | 46 (57.5) |
| Inform respective village or mobile malaria worker to make appointment with the case | | 27 (67.5) | 28 (70.0) | 55 (68.8) |
| Do not revisit the index case | | 2 (5.0) | 0 (0.0) | 2 (2.5) |
| Mark the case as imported | | 0 (0.0) | 1 (2.5) | 1 (1.2) |
| Others | | 1 (2.5) | 0 (0.0) | 1 (1.2) |

## Supplementary table 8: Human resources for case investigation (survey)

|  | **Malaria program stakeholders**  **(n=40)** | **Frontline health workers**  **(n=40)** | **Total**  **(N=80)** |
| --- | --- | --- | --- |
|  | n (%) | n (%) | n (%) |
| **Who is responsible for performing case investigations in your malaria programme?** | | | |
| Village malaria worker or mobile malaria worker | 29(72.5) | *-* |  |
| Others | 11(27.5) | *-* |  |
| **Specify “Others” responsible for performing case investigation in your malaria** | | | |
| Health centre staff | 7(63.7) | *-* |  |
| Provincial, operational district or health centre level staff | 2(18.2) | *-* |  |
| Not mentioned | 2(18.2) | *-* |  |
| **Are you personally involved in malaria case investigations?** | | | |
| Yes, always | - | 35 (87.5) |  |
| Yes, sometimes | - | 4 (10.0) |  |
| No, never | - | 1 (2.5) |  |
| **Do you follow the standard operation procedure for case investigations?** | | | |
| Yes | - | 38 (95.0) |  |
| Missing | - | 2 (5.0) |  |

## Supplementary table 9: Case investigation for classification (survey)

|  | **Malaria program stakeholders**  **(n=40)** | **Frontline health workers**  **(n=40)** | **Total**  **(N=80)** |
| --- | --- | --- | --- |
|  | n (%) | n (%) | n (%) |
| **Does case investigation involve mapping the location of the index case?** | | | |
| No | 14 (35.0) | 13 (32.5) | 27 (33.8) |
| Yes | 26 (65.0) | 27 (67.5) | 53 (66.2) |
| **Does your programme collect information from index cases on their travel history?** | | | |
| No | 2 (5.0) | 6 (15.0) | 8 (10.0) |
| Yes | 38 (95.0) | 34 (85.0) | 72 (90.0) |
| **If answered “Yes”, does your programme collect information on travel within the district of residence?** | | | |
| No | 2 (5.3) | 3 (8.8) | 5 (6.9) |
| Yes | 36 (94.7) | 31 (91.2) | 67 (93.1) |
| **If answered “Yes”, does your programme collect information on travel outside the district of residence?** | | | |
| No | 4 (10.5) | 4 (12.1) | 8 (11.3) |
| Yes | 34 (89.5) | 29 (87.9) | 63 (88.7) |
| Missing | 0 (0) | 1 (2.9) | 1 (1.4) |
| **If answered “Yes”, does your programme collect information on travel outside of the country?** | | | |
| No | 8 (22.2) | 9 (32.1) | 17 (26.6) |
| Yes | 28 (77.8) | 19 (67.9) | 47 (73.4) |
| Missing | 2 (5.3) | 6 (17.6) | 8 (11.1) |
| **How does your programme define imported cases?** | | | |
| Case originated in another country | 30 (75.0) | 24 (60.0) | 54 (67.5) |
| Case occurring within the country but from a different province, district, or other administrative unit | 10 (25.0) | 16 (40.0) | 26 (32.5) |

## Supplementary table 10: Supervision and training for case investigation (survey)

|  | Supervision | | | Training | | |
| --- | --- | --- | --- | --- | --- | --- |
|  | **Malaria program stakeholders**  (n=40) | **Frontline health workers**  (n=40) | **Total**  (N=80) | **Malaria program stakeholders**  (n=40) | **Frontline health workers**  (n=40) | **Total**  (N=80) |
|  | n (%) | n (%) | n (%) | n (%) | n (%) | n (%) |
| Conducting or receiving supervision and training for case investigation | | | | | | |
| No | 7(17.5) | 2(5.0) | 9(11.3) | 0 (0.0) | 0 (0.0) | 0 (0.0) |
| Yes | 33(82.5) | 38(95.0) | 71(88.7) | 40 (100.0) | 40 (100.0) | 80 (100.0) |
| Frequency of supervision and training | | | | | | |
| Monthly | 13 (39.4) | 25 (65.8) | 38 (53.5) | 6 (15.0) | 8 (20.0) | 14 (17.5) |
| Quarterly | 13 (39.4) | 1 (2.6) | 14 (19.7) | 5 (12.5) | 1 (2.5) | 6 (7.5) |
| Yearly | 3 (9.1) | 4 (10.5) | 7 (9.9) | 19 (47.5) | 23 (57.5) | 42 (52.5) |
| Every two years | - | - | - | 10 (25.0) | 8 (20.0) | 18 (22.5) |
| Others | 4 (12.1) | 7 (18.4) | 11 (15.5) |  |  |  |
| Source of supervisor | | | | | | |
| Health centre | - | 30 (79.0) | - | - | - | - |
| Operation district | - | 11 (29.0) | - | - | - | - |
| Provincial health department | - | 5 (13.2) | - | - | - | - |
| Catholic release service organisations | - | 4 (10.5) | - | - | - | - |
| Malaria stakeholder | - | 3 (7.9) | - | - | - | - |

## Supplementary table 11: Foci response activities (survey)

|  | **Malaria program stakeholders**  **(n=40)** | **Frontline health workers**  **(n=40)** | **Total**  **(N=80)** |
| --- | --- | --- | --- |
|  | n (%) | n (%) | n (%) |
| **What kinds of responses may be triggered when a malaria focus is identified?** | | | |
| Raising awareness about malaria transmission | 36(90) | 39(97.5) | 75(93.8) |
| Raising awareness about malaria prevention | 1(2.5) | 30(75) | 31(38.8) |
| Providing additional vector control if needed | 1(2.5) | 24(60) | 25(31.2) |
| Entomological surveillance | 2(5) | 3(7.5) | 5(6.2) |
| Spot check for mosquito breeding ground | 0(0) | 3(7.5) | 3(3.8) |

## Supplementary table 12: Impressions on reactive surveillance and response strategies (survey)

|  | **Malaria program stakeholders**  **(n=40)** | **Frontline health workers**  **(n=40)** | **Total**  **(N=80)** |
| --- | --- | --- | --- |
|  | n (%) | n (%) | n (%) |
| **Are there any RASR activities specifically targeted to mobile and migrant populations, including forest-goers?** | | | |
| No | 19 (47.5) |  |  |
| Yes | 21 (52.5) |  |  |
| **In your experience, does information gained from case and foci investigations and classification influence the RASR activities that are carried out?** | | | |
| No | 27 (67.5) | 30 (75.0) | 57 (71.2) |
| Yes | 13 (32.5) | 10 (25.0) | 23 (28.8) |
| **Do you think current RASR activities are sufficient for targeting *P. vivax* elimination?** | | | |
| No | 15 (37.5) |  |  |
| Yes | 25 (62.5) |  |  |
| **In your experience, has the COVID-19 pandemic had an impact on successful implementation of RASR activities?** | | | |
| No | 17 (42.5) | 27 (67.5) | 44 (55.0) |
| Yes | 23 (57.5) | 13 (32.5) | 36 (45.0) |
