# Supplementary material for: Evaluating malaria reactive surveillance and response strategies in northeast Cambodia: a mixed-methods study
Source: Malar J. 2025 Jul 13;24:229. doi: 10.1186/s12936-025-05475-7 (PMC12255966; doi:10.1186/s12936-025-05475-7)
Supplement: Supplementary file 4 — Additional file 4. Strengthening the Reporting of Observational Studies in Epidemiology checklist [file 12936_2025_5475_MOESM4_ESM.docx]

**Additional file 4: STROBE checklist - Strengthening the Reporting of Observational Studies in Epidemiology checklists**

STROBE Statement—checklist of items that should be included in reports of cross-sectional studies

|  | Item No. | Recommendation | Page  No. | Relevant text from manuscript |
| --- | --- | --- | --- | --- |
| **Title and abstract** | 1 | (*a*) Indicate the study’s design with a commonly used term in the title or the abstract | 1 | Evaluating malaria reactive surveillance and response strategies in northeast Cambodia:  A mixed-methods study |
|  |  | (*b*) Provide in the abstract an informative and balanced summary of what was done and what was found | 2-3 | A mixed-methods study of secondary data analysis of aggregated routine malaria dataset, and cross-sectional survey, in-depth interviews and focus group discussions with malaria program stakeholders, frontline health workers and mobile and migrant populations was conducted in Ratanakiri and Stung Treng provinces. Quantitative and qualitative data were analysed descriptively and thematically, and then triangulated for interpretation.  In 2020 and 2022, 72% and 59% of malaria cases in Ratanakiri and Stung Treng Provinces were notified and investigated within one day after diagnosis. Reactive case detection involved screening of family members, 20 neighbouring households or populations within the one-kilometre geographic radius around the index case for secondary malaria cases showing the timeliness of 89% and 45% in Ratanakiri and Stung Treng, respectively in both 2020 and 2022. Despite having challenges including less community participation in reactive case detection, poor mobile phone network coverage and road conditions, a heavy workload at the commune health centre level, and inadequate technical knowledge among village malaria workers and insufficient budget to execute RASR, the existing RASR strategy was deemed acceptable among all levels of health personnels. |
| Introduction | | | |  |
| Background/rationale | 2 | Explain the scientific background and rationale for the investigation being reported | 3-4 | Countries in the Greater Mekong Subregion (GMS) including Cambodia aim to eliminate malaria latest by 2030 and have made significant progress towards the goal over the past decades.  Cambodia adopted the 1-3-7 approach as its RASR strategy after pilot-testing in Sampov Loun operational district in 2015 for its implementation feasibility [7]. According to surveillance guidelines for malaria elimination by the National Centre for Parasitology, Entomology, and Malaria Control (CNM), the strategy involves case notification, investigation, and classification and reporting to the electronic Malaria Information System on the same day as diagnosis, reactive case detection (RACD) within three days of the notification and classification, and investigation and classification of every new active focus within one week [8].  To date, there has been no comprehensive and systematic evaluation of Cambodia’s RASR strategy in terms of its timeliness, acceptability, and facilitators and barriers of implementation. |
| Objectives | 3 | State specific objectives, including any prespecified hypotheses | 4 | This mixed-methods study investigates how Cambodia implements its RASR strategy, its performance in terms of timeliness, and facilitator and barriers, and acceptability of implementation of the RASR strategy in its remote provinces, Ratanakiri and Stung Treng, to provide recommendations on how the strategy may be improved in the context of existing national health system |
| Methods | | | |  |
| Study design | 4 | Present key elements of study design early in the paper | 4-5 | The mixed-methods study included secondary data analysis of aggregated datasets for timeliness of RASR activities in Ratanakiri and Stung Treng Provinces in Cambodia reported between 1st January 2020 and 30th September 2022 (Additional file 1), quantitative cross-sectional survey, in-depth interviews (IDI) and focus group discussions (FGD). |
| Setting | 5 | Describe the setting, locations, and relevant dates, including periods of recruitment, exposure, follow-up, and data collection | 5 | Ratanakiri and Stung Treng Provinces (Figure 1) are remote provinces in northeastern Cambodia bordering Lao People’s Democratic Republic and Vietnam, characterized by tropical forests, rubber plantations and fallow lands. Ethnically diverse and home to mobile and migrant populations (MMPs), these provinces are among the highest risk areas for malaria in Cambodia.  The aggregated dataset for secondary data analysis was extracted from the National Malaria Surveillance Database of CNM on 2nd November 2022.  The survey, FGDs and IDIs were conducted in person in Khmer language between August 2022 and March 2023. |
| Participants | 6 | *Cross-sectional study*—Give the eligibility criteria, and the sources and methods of selection of participants | 5-7 | Participants were selected purposively in consultation with CNM and in-country malaria implementing partners based on the criteria of having experiences with the RASR strategy and malaria program, and willingness to participate in the study. |
| Variables | 7 | Clearly define all outcomes, exposures, predictors, potential confounders, and effect modifiers. Give diagnostic criteria, if applicable |  | Timeliness of case notification: Number of malaria cases notified and classified on the same day as diagnosis.  Timeliness of RACD: Number of malaria cases for which RACD was completed within three days.  Timeliness of focus investigation: Number of malaria cases for which focus investigation was completed within one week.  Minimum number of households for RACD: minimum number of households around a positive index case screened during RACD  Minimum geographic radius for RACD: minimum geographic radius in kilometre around a positive index case screened during RACD  Barriers of RASR activities: barriers encountered by the malaria program stakeholders and frontline health workers implementing RASR activities in the study provinces. |
| Data sources/ measurement | 8* | For each variable of interest, give sources of data and details of methods of assessment (measurement). Describe comparability of assessment methods if there is more than one group |  | Secondary data were obtained from the National Malaria Surveillance Database of CNM and the surveys were conducted using questionnaires for malaria program stakeholders of managerial level and frontline health workers providing malaria service in the field. |
| Bias | 9 | Describe any efforts to address potential sources of bias |  | Selection criteria were clearly defined by the CNM and its implementing partners. The participants were selected from different districts or villages of the two study provinces. Source of information ranged from village malaria workers to provincial level managerial staff. Reasons for not participating in the study were documented. Information acquired from the survey were triangulated with that of secondary data and qualitative data. |
| Study size | 10 | Explain how the study size was arrived at |  | 40 malaria program stakeholders and 40 frontline health workers were selected for the surveys. |

Continued on next page

| Quantitative variables | 11 | Explain how quantitative variables were handled in the analyses. If applicable, describe which groupings were chosen and why |  | Quantitative categorical variables in the survey were predefined in close-ended question. Open ended numerical variables (e.g., minimum radius of RACD) were categorised according to the responses because some participants responded the radius in range while some responded exact size of the radius. |
| --- | --- | --- | --- | --- |
| Statistical methods | 12 | (*a*) Describe all statistical methods, including those used to control for confounding | 6-7 | From the secondary dataset, the percentage of timely malaria case notification and classification, RACD and focus investigation were calculated using the formulae: number of cases notified and classified on the same day as diagnosis divided by total number of malaria cases, number of cases for which RACD was completed within three days divided by total number of eligible cases for RACD, and number of cases for which focus investigation was completed within one week divided by number of eligible cases for focus investigation. The findings were multiplied by 100 to get the percentages.  The datasets were then imported into R version 4.2.1 for analysis including generating frequencies and percentages for categorical variables and calculating medians and interquartile ranges for numerical variables. |
|  |  | (*b*) Describe any methods used to examine subgroups and interactions | NA | NA |
|  |  | © Explain how missing data were addressed | NA | Missing values were minimised by carefully conducting the survey so that there were no missing values for the outcome variables. |
|  |  | (*d*) *Cohort study*—If applicable, explain how loss to follow-up was addressed  *Case-control study*—If applicable, explain how matching of cases and controls was addressed  *Cross-sectional study*—If applicable, describe analytical methods taking account of sampling strategy | NA | NA |
|  |  | (*e*) Describe any sensitivity analyses | NA | NA |
| Results | | | | |
| Participants | 13* | (a) Report numbers of individuals at each stage of study—eg numbers potentially eligible, examined for eligibility, confirmed eligible, included in the study, completing follow-up, and analysed |  | Additional file 4, Supplementary table 1 |
|  |  | (b) Give reasons for non-participation at each stage | NA | NA |
|  |  | © Consider use of a flow diagram | NA | NA |
| Descriptive data | 14* | (a) Give characteristics of study participants (eg demographic, clinical, social) and information on exposures and potential confounders |  | Additional file 4, Supplementary table 1 |
|  |  | (b) Indicate number of participants with missing data for each variable of interest | NA | NA |
| Outcome data | 15* |  |  |  |
|  |  |  |  |  |
|  |  | *Cross-sectional study—*Report numbers of outcome events or summary measures | 8, 11, 12 | Around 72% of cases in Ratanakiri and 59% of cases in Stung Treng were notified and classified on the same day as diagnosis in 2020 and 2022.  The timeliness of RACD was similar in 2020 and 2022 (but varied according to province Ratanakiri, 89%; Stung Treng, ~45%) and was lowest in 2021 (73% and 24% in Ratanakiri and Stung Treng respectively) (Table 1).  Majority of the survey participants reported that RACD always screened the household members of the index cases (92.5%, 74/80) and included all the household members (92.3%, 72/78). They reported that RACD also included screening both asymptomatic and febrile neighbours (81%, 65/80; Table 3). The median number of households screened was 20 (min-max: 3-40, n=78). Most survey participants reported that minimum screening radius was within one kilometre around a positive index case (83%, 55/80; Table 3). IDIs revealed that co-travellers of the index cases were also tested during the RACD.  Secondary data analysis revealed that the majority of the eligible foci with local *P. falciparum* or mixed cases were investigated and classified within one week (71%, 41/58) after a positive malaria case had been reported. Notably, there were zero eligible cases for focus investigation in Ratanakiri Province in 2020 and 2022. In 2021, exactly half of the foci in Ratanakiri (50%, 5/10) were investigated within seven days. Stung Treng province achieved >90% timeliness in focus investigation in 2020 and 2022 but was lower in 2021 (18%, 2/11) (Table 1).  Subsection - Acceptability of RASR strategy,  Subsection - Feasibility of RASR strategy  Subsection - Challenges for successful implementation of RASR strategy |
| Main results | 16 | (*a*) Give unadjusted estimates and, if applicable, confounder-adjusted estimates and their precision (eg, 95% confidence interval). Make clear which confounders were adjusted for and why they were included | NA | NA |
|  |  | (*b*) Report category boundaries when continuous variables were categorized | NA | NA |
|  |  | (*c*) If relevant, consider translating estimates of relative risk into absolute risk for a meaningful time period | NA | NA |

Continued on next page

| Other analyses | 17 | Report other analyses done—eg analyses of subgroups and interactions, and sensitivity analyses | NA | NA |
| --- | --- | --- | --- | --- |
| Discussion | | | | |
| Key results | 18 | Summarise key results with reference to study objectives | 19 | There were delays in case classification and notification in about 30-40% of cases as well as RACD and focus investigation in 2020 and 2022 which was amplified in 2021. Study participants acknowledged Cambodia’s adoption of the 1-3-7 approach and found it to be acceptable and feasible to implement in the study provinces. Nevertheless, poor community participation, insufficient financial support, telecommunication problems for reporting and contacting cases, and transportation difficulties in remote areas were identified as major challenges to successful implementation of the RASR strategy. These barriers must be addressed so that the timely execution of each step of RASR will be improved and achieve 100%, a global standard in the malaria elimination program, which will ultimately contribute towards achieving national and regional malaria elimination goals. |
| Limitations | 19 | Discuss limitations of the study, taking into account sources of potential bias or imprecision. Discuss both direction and magnitude of any potential bias | 25 | However, the study was conducted only in Ratanakiri and Stung Treng provinces and generalization of the study findings in other provinces should be made with caution. Furthermore, only the aggregated secondary dataset that lacks individual line lists of malaria patients was extracted from Malaria Information System and analysed. Thus, factors influencing the timeliness of the RASR activities could not be further explored. |
| Interpretation | 20 | Give a cautious overall interpretation of results considering objectives, limitations, multiplicity of analyses, results from similar studies, and other relevant evidence | 18-25 | Discussion |
| Generalisability | 21 | Discuss the generalisability (external validity) of the study results | 27 | However, the study was conducted only in Ratanakiri and Stung Treng provinces and generalization of the study findings in other provinces should be made with caution. |
| Other information | |  | | |
| Funding | 22 | Give the source of funding and the role of the funders for the present study and, if applicable, for the original study on which the present article is based | 36,37 | This study was funded by an international funding organisation (Grant Number: QSE-M-UNOPS-BI-20864-007-61) to all authors and the National Health and Medical Research Council of Australia (Leadership Fellowship and Centre for Research Excellence) awarded to FJIF and WHO. The Burnet Institute is funded by a Victorian State Government Operational Infrastructure Support grant. Investigators from National Centre for Parasitology, Entomology and Malaria Control are government staff, and their salaries and infrastructure are contributed by the Cambodia Ministry of Health. The funders did not have input on the design, collection, analysis, interpretation and publication of the study results. |

*Give information separately for cases and controls in case-control studies and, if applicable, for exposed and unexposed groups in cohort and cross-sectional studies.

**Note:** An Explanation and Elaboration article discusses each checklist item and gives methodological background and published examples of transparent reporting. The STROBE checklist is best used in conjunction with this article (freely available on the Web sites of PLoS Medicine at http://www.plosmedicine.org/, Annals of Internal Medicine at http://www.annals.org/, and Epidemiology at http://www.epidem.com/). Information on the STROBE Initiative is available at www.strobe-statement.org.
